# Abundance trends for river macroinvertebrates vary across taxa, trophic group and river typology

**DOI:** 10.1111/gcb.16549

**Published:** 2022-12-15

**Authors:** Kathryn E. Powell, Tom H. Oliver, Tim Johns, Manuela González‐Suárez, Judy England, David B. Roy

**Affiliations:** ^1^ UK Centre for Ecology and Hydrology Wallingford UK; ^2^ School of Biological Sciences University of Reading Reading UK; ^3^ Environment Agency Wallingford UK

**Keywords:** abundance trends, biodiversity change, ecological status, ecosystem function, freshwater macroinvertebrates, insect declines, river macroinvertebrates, river typology, spatial heterogeneity

## Abstract

There is mounting evidence that terrestrial arthropods are declining rapidly in many areas of the world. It is unclear whether freshwater invertebrates, which are key providers of ecosystem services, are also declining. We addressed this question by analysing a long‐term dataset of macroinvertebrate abundance collected from 2002 to 2019 across 5009 sampling sites in English rivers. Patterns varied markedly across taxonomic groups. Within trophic groups we detected increases in the abundance of carnivores by 19% and herbivores by 14.8%, while we estimated decomposers have declined by 21.7% in abundance since 2002. We also found heterogeneity in trends across rivers belonging to different typologies based on geological dominance and catchment altitude, with organic lowland rivers having generally higher rates of increase in abundance across taxa and trophic groups, with siliceous lowland rivers having the most declines. Our results reveal a complex picture of change in freshwater macroinvertebrate abundance between taxonomic groups, trophic levels and river typologies. Our analysis helps with identifying priority regions for action on potential environmental stressors where we discover macroinvertebrate abundance declines.

## Graphical abstract

We addressed the question of whether freshwater invertebrates, which are key providers of ecosystem services, are declining in abundance by analysing a long‐term dataset of macroinvertebrates in English rivers. We found a complex picture of change in abundance between taxonomic groups, trophic levels and river typologies. Within trophic groups we detected 19% increases in carnivore abundance, and 14% for herbivores, while estimating decomposers declined by 21.7% since 2002. Trends are heterogeneous across rivers of different typologies, organic lowland rivers having generally higher rates of increase in abundance across taxa and trophic groups, and siliceous lowland rivers having the most declines.

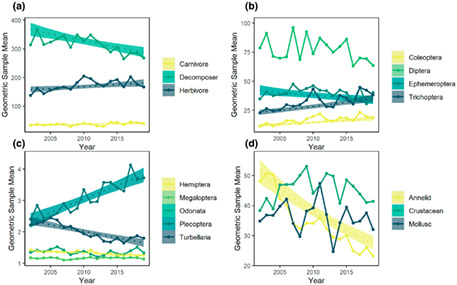

## 1 INTRODUCTION

Biodiversity is rapidly changing across the globe (Díaz et al., 2019). Long‐term datasets suggest widespread declines in richness, abundance and biomass of terrestrial insects and other arthropods, including steep declines in biomass of flying insects in areas of Europe (Didham et al., 2020; Hallmann et al., 2017, 2019; Wagner et al., 2021). The spatial and taxonomic extent of these declines are unclear, as well as whether declines are spread across both terrestrial and freshwater systems, and this is further complicated by reported abundance and biomass increases across several taxa at the local scale (Crossley et al., 2020).

Declines in arthropod abundance could have negative consequences on ecosystems, as these taxa underpin vital ecosystem functions and services (Noriega et al., 2018; Schowalter et al., 2018). Freshwater macroinvertebrates provide a range of key ecological functions and associated ecosystem services in both freshwater and terrestrial systems (Macadam & Stockan, 2015). For example, benthic invertebrates constitute a significant part of the diet of a range of fish, bird and mammal species (e.g. Michel & Overdorff, 1995). Burrowing and sedentary macroinvertebrates create structural habitat complexity, benefitting other invertebrate and fish species (Covich et al., 1999). Macroinvertebrate communities are also essential regulators of nutrient cycles in freshwater ecosystems (Cuffney et al., 1990; Hieber & Gessner, 2002), with the activity of detritivorous macroinvertebrates, such as ‘shredders’ and ‘grazers’, being essential for breaking down organic matter such as leaf litter entering streams from riparian habitats (Graça, 2001). This process, along with herbivorous consumption of macrophytes, stimulates the transfer of nutrients to other organisms, thereby regulating the water self‐purification of freshwater systems and supporting diverse and complex food webs (Graça, 2001; Wallace & Webster, 1996). The reduction of macroinvertebrate abundance across different taxa and feeding groups will likely have negative consequences for these ecosystem functions and services, in particular given that ecosystem functions are largely driven by the abundance of common species (Winfree et al., 2015).

Biodiversity change in freshwater macroinvertebrate communities has previously been explored in terms of species richness, prevalence, occurrence and distribution changes (e.g., Environment Agency, 2021; Fried‐Petersen et al., 2020; Jourdan et al., 2018; Outhwaite et al., 2020; Vaughan & Ormerod, 2012a, 2012b). In contrast to the commentary on terrestrial species, taxonomic richness and prevalence (the number of species within families) as well as occurrence (the presence of species across space) of freshwater macroinvertebrates has been found to have increased over the last two decades in some areas, such as Great Britain (Outhwaite et al., 2020; Vaughan & Ormerod, 2012b). This has been largely attributed to water quality improvements, such as a decrease in phosphate load and catchment acidification from very poor levels before the 1990s, despite other pressures on freshwater ecosystems, such as climate change, intensifying over the same time period (Vaughan & Gotelli, 2019; Vaughan & Ormerod, 2012a, 2012b; Whelan et al., 2022). Other reported trends in freshwater macroinvertebrates, however, are complex, and are thought to be driven by a range of environmental pressures beyond climate change and water quality alone, such as catchment and floodplain land use change and intensification, habitat modification (both the surrounding terrestrial habitats and within the river banks and bed), and flow regulation (Domisch et al., 2011; Feld & Hering, 2007). The fact that freshwater ecosystems are likely highly susceptible to multiple stressors makes untangling trends over space and time, at the hands of a few select environmental drivers, particularly challenging (Leps et al., 2015), with different stressors changing in relative importance depending on the scale of the study (Feld & Hering, 2007; Sundermann et al., 2013).

A meta‐analysis of invertebrate trends across continents (van Klink et al., 2020) revealed differences between freshwater and terrestrial abundance, with the former increasing. However, this study did not explore underlying differences among taxa or across space (Desquilbet et al., 2020; Jähnig et al., 2020). Overlooked heterogeneity can mask local patterns that affect the provision of important ecosystem functions and services. Heterogeneity in trend patterns may partly be explained by underlying hydrological, geological and geographical conditions, which constitute ‘river typology’. A ‘typological approach’, as we use in this study, categorizes rivers, based on the underlying geology around sites and catchment altitude. Using river typologies allows for a more holistic consideration of the environment and the many interacting drivers of community change, as different river types capture broadly different conditions and pressures in freshwater ecosystems (Lyche Solheim et al., 2019; Schmitt et al., 2011). For example, the geological conditions at sites generally affect the filtration of pollutants into rivers and the way in which rivers are fed, which could influence the severity of the environmental pressures on freshwater ecosystems (Berrie, 1992). Calcareous rivers are usually fed by groundwater sources, the water having filtered through more porous sediment (limestone and chalk), whereas rivers dominated by other geological sediment types (siliceous and organic peat rivers) tend to be surface water fed (Berrie, 1992). Surface water is more susceptible to flow changes and surface conditions, which can exacerbate the effects of warming water temperatures and nutrient inputs when at low flow, as well as affect colonization rates of macroinvertebrates when at higher flows (Eveleens et al., 2019; Ledger & Milner, 2015; Mosley, 2015; Piniewski et al., 2017). Other typological features, such as altitude, may influence abundance trends of macroinvertebrates, given that the uplands are generally more vulnerable to climate warming effects than lowland rivers (Orr et al., 2008; Worrall et al., 2004). On the other hand, lowland rivers often flow through urban areas and may be more susceptible to other pressures on freshwater ecosystems such as the disruption of food webs by invasive species (Mathers et al., 2016), which have increased over recent decades, in lowland rivers of England (Johns et al., 2018). Understanding where abundance of important invertebrates has declined, including freshwater macroinvertebrates, has been hampered by a lack of long‐term data from standardized monitoring schemes (Isaac & Pocock, 2015; Powney et al., 2015; Thomas et al., 2019). Long‐term trends in large systems are also difficult to characterize with statistical confidence as sampling effort is often limited compared to the system scale, causing high fluctuation in interannual variation (Cauvy‐Fraunié et al., 2020). An exception is abundance data for riverine freshwater macroinvertebrates collected over multiple decades by the Environment Agency (EA); the government authority responsible for monitoring the health and water quality of freshwaters in England. These data have primarily been used for the qualitative determination of environmental quality across waterbodies and catchments, in alignment with monitoring requirements, such as for the European Union Water Framework Directive (WFD, 2000).

Here, we realize the potential of this dataset to identify long‐term abundance changes for freshwater macroinvertebrates across diverse rivers and regions in England. We use the dataset to characterize and compare trends in: (1) the abundance of different taxonomic groups (at family level and above) of riverine macroinvertebrates, (2) the abundance change of different trophic groups, to shed light on the potential functional changes within rivers and (3) the spatial pattern of long‐term trends across different types of river.

## 2 METHODS

### 2.1 Macroinvertebrate abundance

Abundance data for riverine macroinvertebrates in England were extracted from the EA's ecological monitoring database (Environment Agency, 2020a). The data were filtered to only include 3‐min kick‐sample data as the primary method for sampling freshwater invertebrates (approximately 99% of samples). Three‐minute kick samples are a standardized, internationally recognized, semi‐quantitative approach to assessing macroinvertebrate ecology and water quality in rivers using invertebrate diversity indicators (Furse et al., 1981; Murray‐Bligh, 1999).

Prior to the implementation of the European Union Water Framework Directive in 2000 (WFD, 2000), abundance estimates were based on categories (0–9, 10–99, 100–999 etc.). In 2002, the EA started recording more exact abundance estimates and enacting improved quality control procedures, whereby one in every 10 samples were independently re‐analysed. Hence, although the original dataset covered sampling years from 1991, our analysis was restricted to the years 2002–2019.

Data were further filtered to only include sites sampled for a minimum of 3 years out of a total of 18 in both spring (March–May) and autumn (September–November) to avoid seasonal bias. In order to test whether this was an appropriate minimum time series length to use in our models, we ran equivalent analyses with sites sampled in both seasons for a minimum of 10 years (see Figures S1–S3). Trends across the two datasets were significantly positively correlated (Pearson's correlation coefficient, *r* = .83), but limiting the dataset to sites sampled across a minimum of 10 years in both seasons greatly reduced the number of sites across river typology categories. This has the potential to introduce spatial bias into our models and, therefore, we report on the more extensive dataset analysis.

**FIGURE 1:**
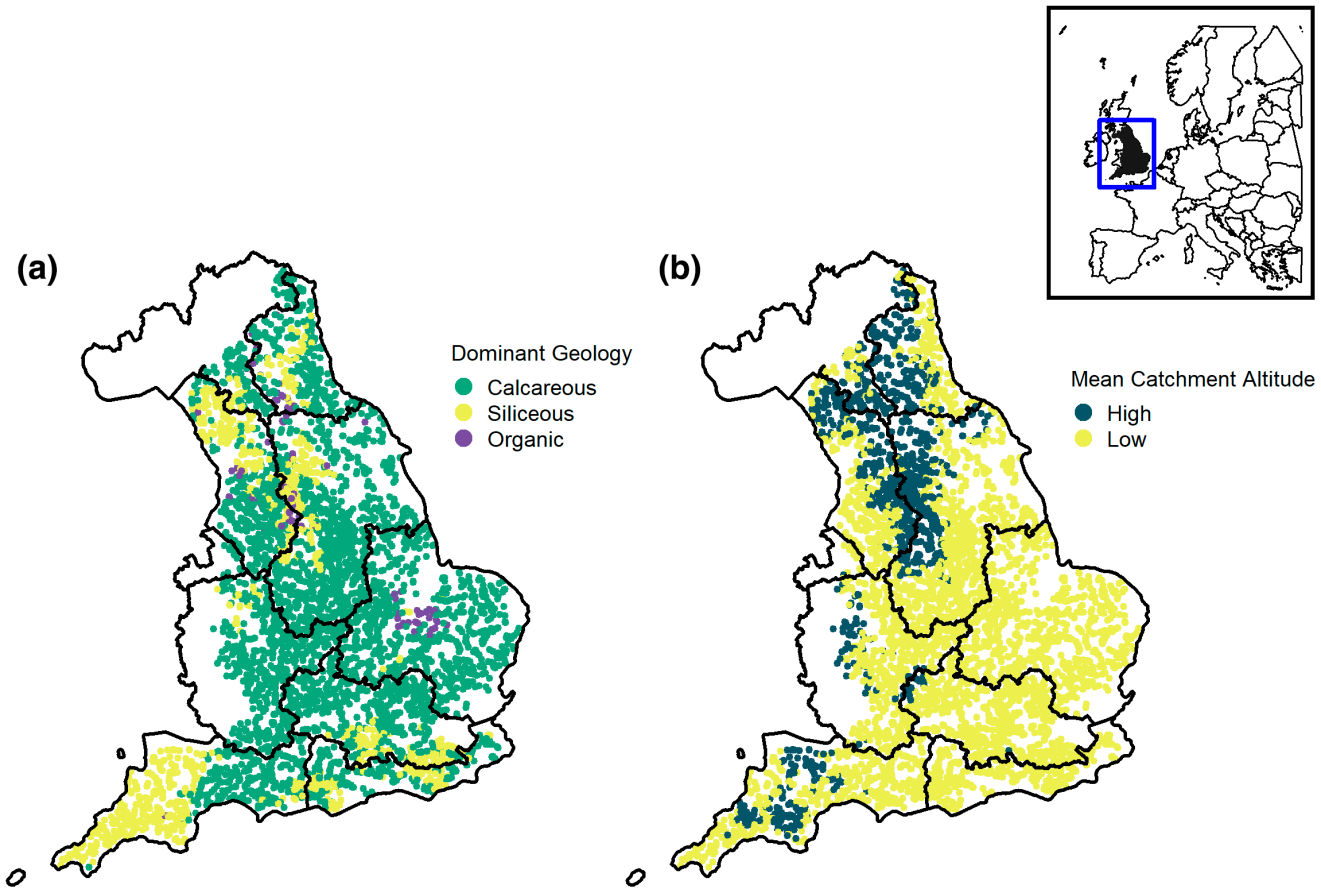
Map of the site locations in England (*n* = 5009) selected for mixed models, coloured by river typology (*n* = 6; (a) three dominant geological substrate types—Calcareous, organic and siliceous, and (b) two mean catchment altitude categories—High and low). The number of sites within each typology is as follows; calcareous high: 525, calcareous low: 3289, organic high: 72, organic low: 45, siliceous high: 525, siliceous low: 553. Map lines delineate study areas and do not necessarily depict accepted national boundaries.

After filtering the dataset according to these criteria, our final dataset from 2002 to 2019 included 67,757 individual macroinvertebrate samples from 5009 sites (out of 10,136 sites in the original dataset). This equates to an average of 3764 samples a year, covering 2774 waterbodies distributed across the 10 river basins defined under the European Union Water Framework Directive in England: Anglian, Humber, North West, Northumbria, Severn, Solway Tweed, South East, South West and Thames (Figure 1). The final dataset provides a wide national distribution of sites representative of the main river conditions, albeit with a bias towards mid to lower perennial reaches (reflective of the purpose of the monitoring programmes instigated for environmental quality monitoring, rather than a river's intrinsic biodiversity).

### 2.2 Taxonomic groups

The identification of macroinvertebrates in the database, including within individual samples, is given at a mixture of taxonomic levels, meaning species‐level trends in abundance change could not be calculated due to a lack of consistency between and within samples. Instead, we pooled and analysed the data at two different levels: (1) wider taxonomic groups (non‐insect freshwater macroinvertebrates: annelids, molluscs, Turbellaria and crustaceans, and individual insect orders: Ephemeroptera, Trichoptera, Plecoptera, Coleoptera, Diptera, Megaloptera, Hemiptera and Odonata); and (2) taxonomic families, representing observations for which this level of identification was available.

### 2.3 Trophic groups

We also pooled and analysed data considering main trophic groups (carnivores, herbivores and decomposers). We allocated macroinvertebrate dietary preferences for each genus where this level of identification was given in the dataset, according to the main food source described in the functional and morphological traits database for European freshwater macroinvertebrates (Tachet et al., 2010). Tachet et al. (2010) use a fuzzy‐coded system whereby dietary components are given a score between 0 and 5 describing the affinity for the following dietary components: ‘microorganisms’, ‘detritus < 1 mm’, ‘dead plant ≥ 1 mm’, ‘living microphytes’, ‘living macrophytes’, ‘dead animal ≥ 1 mm’, ‘living microinvertebrates’, ‘living macroinvertebrates’ and ‘vertebrates’. In most cases, abundance data were entered at the family or higher taxonomic group level in the EA database; for those cases, diet scores were estimated as weighted means of the diet score data (values between 0 and 5) for that grouping, weighting based on the relative abundance of taxa identified in our abundance dataset. Hence, taxa included in Tachet et al. (2010) but not recorded in rivers in England by the EA data were excluded when calculating average family or group dietary scores, and more common and abundant genera had a proportionally greater influence over average dietary scores. This allowed the final diet scores at the group level to reflect the probability of the individual identified at this higher level possessing a particular dietary trait. Genera, families and higher taxonomic groupings were allocated to trophic groups based on items with highest dietary scores: carnivores had highest scores for ‘vertebrates’, ‘living macroinvertebrates’, ‘living microinvertebrates’; herbivores had highest scores for ‘living macrophytes’ or ‘living microphytes’; and decomposers had highest scores for ‘dead plant ≥1 mm’, ‘dead animal ≥1 mm’ and ‘detritus’. Freshwater invertebrates could be included in more than one trophic group if distinct diet items had equally high scores (as may occur in omnivores). No genera, family or group in our abundance dataset had highest dietary scores (preference) for microorganisms.

### 2.4 River typology

To categorize sampling sites by typology, we used criteria from the EU Water Framework Directive's descriptions of river typologies (Water Framework Directive UKTAG, 2003), including the dominant geology at the site and mean catchment altitude. We used river typology data held by UK Centre for Ecology and Hydrology and used for the River Invertebrate Classification Tool (RICT, Scottish Environment Protection Agency) which modelled the proportion of different sediments (chalk, limestone, clay, hard rock and peat) located along rivers to calculate the dominant geological sediment at sampling sites. Where sites were dominated by chalk or limestone, sites were classified as ‘Calcareous’. Where the dominant sediment type was clay or hard rock, sites were classified as ‘Siliceous’. We classified sites dominated by peat as ‘Organic’. Thirty‐eight sites were excluded from the analysis, due to missing or multiple dominant geologies in the RICT typology data. Sites were also grouped by mean catchment altitude: mean altitudes ≥200 m were categorized as ‘high’, and <200 m as ‘low’. The combination of these classifications resulted in six river typologies for our analyses: ‘Calcareous/High Altitude’, ‘Calcareous/Low Altitude’, ‘Organic/High Altitude’, ‘Organic/Low Altitude’, ‘Siliceous/High Altitude’ and ‘Siliceous/Low Altitude’ (Table 1).

**TABLE 1:** Criteria used for categorizing sites by river typology

Type	Dominant geology	Mean catchment altitude (m)	Number of sites
I	Calcareous	≥200 (High)	525
II	Calcareous	<200 (Low)	3289
III	Organic	≥200 (High)	72
IV	Organic	<200 (Low)	45
V	Siliceous	≥200 (High)	525
VI	Siliceous	<200 (Low)	553

### 2.5 Statistical analysis

To test whether macroinvertebrate abundance changed over time on a national scale, we fitted hierarchical generalized linear mixed regression models (GLMMs; Bates et al., 2015) for various response variables calculated as the sum of counts per sample for the three aggregated groups (wider taxonomic groups, taxonomic families and trophic groups). Poisson GLMMs were chosen to fit the left‐skewed count data, where there were high frequencies of low abundances within groups. For all three aggregated datasets (wider taxonomic groups, taxonomic families and trophic groups) we fitted a national‐level model with year as the sole fixed factor to describe general patterns. For the wider taxonomic group and trophic group datasets we additionally fitted a river typology model including year, river typology and their interaction as fixed factors to explore trend variation among typologies. In all models, to facilitate interpretation, year was converted to an integer from 0 to 17, with 0 representing 2002 and 17 corresponding to 2019. In both models the random effects structure included: random intercepts and slopes for each site to account for spatial pseudoreplication and within‐site variation in temporal trends; random intercepts for year to account for within‐year pseudoreplication (Daskalova et al., 2021); and random intercepts for each observation to account for non‐zero‐inflated over‐dispersion of counts in the data (Harrison et al., 2018). The use of ‘year’ in both the fixed and random effects of the model allowed us to examine the influence of increasing years on abundance of macroinvertebrates, while reducing the impact of ‘particularly good’ or ‘particularly bad’ years for macroinvertebrates and decreasing the chance of identifying significant trends driven by outlier effects.

We evaluate models using Akaike information criterion (AIC) and Bayesian information criterion (BIC) and tested for differences in abundance trends at the river typology level using analysis of variance tests (ANOVA, Figure S4 and Table S1). AIC was used over AICc due to adequate sample size and a corresponding reduced likelihood of overfitting. Models were fitted with the *lme4* package in R (Bates et al., 2015) and we used the ‘*ggeffects*’ R package (Lüdecke, 2018) to get predicted values for each year from which we calculated overall percentage change (Ѱ) and annual growth rate (AGR) as:

(1) $$Ѱ=\left(\left(y_{n}-y_{1}\right)/y_{1}\right)\times 100$$

and

(2) $$\mathrm{AGR}=Ѱ/\left(n-1\right)$$

where *y*_*n*_ is the model estimate of the abundance value for the final year of the time series (2019), *y*_1_ is the estimated abundance value for the starting year of the time series (2002) and *n* is the number of years total in the time series.

In addition to linear models, we explored potential non‐linear patterns at the national scale (rather than in different river types) using multi‐level hierarchical generalized additive mixed effect models (GAMMs) using the ‘mgcv’ package in R (Wood, 2022). For these models we used the same modelling format expressed above for GLMMs, using the function gam() to include year as a smoothed fixed effect, and random smooths at the site and observation level. We focus on the GLMM format to report our results, in order to calculate and compare changes in abundance and annual growth rates in a consistent way across taxon and trophic groups. The results of these additional analyses are included in the Supporting information (Figure S5 and Table S5).

As sampling effort is not typically uniform across years and river typology, we explored temporal patterns of sampling effort within and across sites and rivers of different typologies (see Figures S1 and S2). Changes in sampling effort between years did not correspond to changes in macroinvertebrate abundance, which varied between different groups. We found no significant effect of total samples taken across river types on macroinvertebrate abundance trends (*β* = −.00495, SE = 0.003, df = 88; *p* > .05; Figure S3).

All statistical analyses were completed in R (version 4.0.0) (R Core Team, 2020).

## 3 RESULTS

### 3.1 National trends

#### 3.1.1 Taxonomic group abundance

**FIGURE 2:**
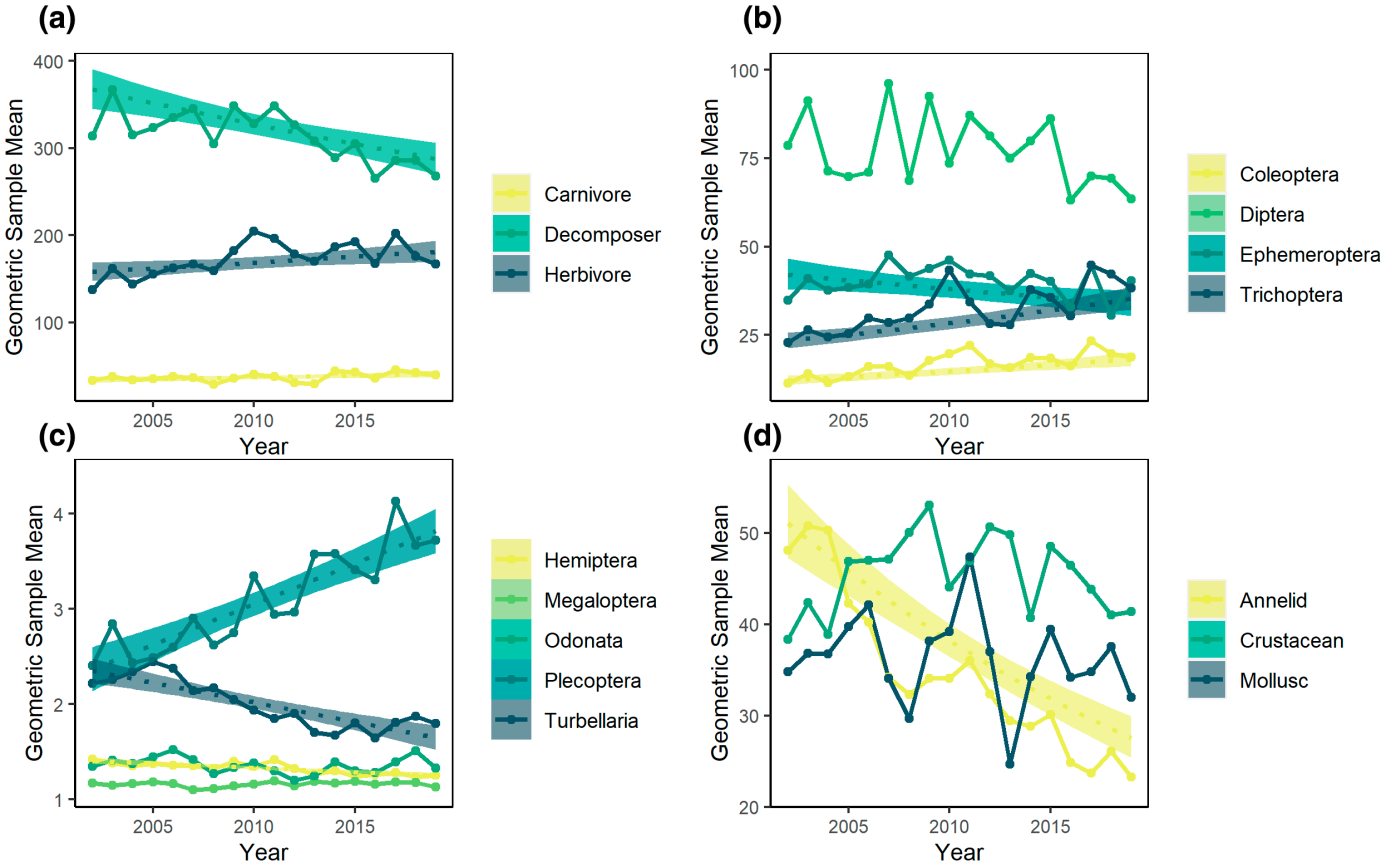
Abundance of river macroinvertebrates from 2002 to 2019 for groups: (a) trophic groups: Carnivores, herbivores and decomposers; (b) insect groups of high mean abundance: Coleoptera (beetles), Diptera (true flies), Ephemeroptera (mayfly), Trichoptera (caddisfly); (c) insect and other invertebrate groups of low mean abundance: Hemiptera (true bugs), Megaloptera (alderfly), Odonata (dragonfly and damselfly), Plecoptera (stonefly) and Turbellaria (flatworms); and (d) other invertebrate groups, of higher mean abundance: Annelids (segmented worms), crustaceans and Molluscs. Abundance is presented as the geometric mean, shown with a solid line. Dashed lines show the model predictions based on the raw data for groups where the effect of ‘year’ on abundance was significant (*p* ≤ .05), with shaded envelopes indicating 95% confidence intervals.

Across the taxonomic groups we studied, we found large differences in baseline abundance values that reflect the relative proportion of these groups living in freshwater ecosystems. The highly abundant groups include annelids, crustaceans, molluscs, Coleoptera, Ephemeroptera and Trichoptera. Groups with low baseline abundance in samples include Plecoptera, Megaloptera, Odonata, Hemiptera and Turbellaria. The difference between these baseline abundances can be explored through the geometric mean values presented in Figures 2 and 4.

We found major differences in the national‐scale abundance trends among the 12 macroinvertebrate taxonomic groups evaluated (Figure 2b–d, Table 2). Among non‐insect macroinvertebrates, we found large declines in annelids and Turbellaria, resulting in 46% and 51.8% total abundance loss respectively over the 18‐year period (Table 2). In contrast, the abundance of crustaceans and molluscs remained largely stable (Figure 2d). Similarly, abundance trends differed among insect orders. Trichoptera, Plecoptera and Coleoptera showed estimated increases of 50.8%, 142.1% and 48.6% respectively over the 18‐year period (Table 2). Trends for Diptera, Hemiptera, Megaloptera and Odonata were stable, while Ephemeroptera significantly decreased in abundance, by an estimated 19.5% over the time period we studied (Figure 2b,c, Table 2).

**TABLE 2:** Summary of coefficients for fixed effects (year), and random effects variance, of generalized linear mixed models of macroinvertebrate abundance including year as a fixed effect. Significant trends (*p*  < .05) are highlighted in bold text. ‘AGR (%)’ = annual growth rate (%), and ‘Total change’ = Total percentage change over the 18‐year time period

Taxonomic group	Intercept		Slope			Random effects (*θ*)				AGR (%)	Total change (%)
	Estimate	SE	Estimate	SE	*p* value	Site (intercept)	Site (slope)	Year (intercept)	OLRE		
Carnivore	3.513	0.057	0.01	0.005	<.05	1.072	0.061	0.111	0.881	1.06	19
Herbivore	5.053	0.037	0.008	0.003	<.05	1.059	0.057	0.066	0.867	0.82	14.8
Decomposer	5.921	0.034	−0.014	0.003	<.001	0.832	0.052	0.062	0.83	−1.21	−21.7
Annelid	3.97	0.043	−0.036	0.004	<.001	1.122	0.07	0.079	1.068	−2.56	−46
Coleoptera	2.477	0.058	0.023	0.005	<.001	2.038	0.104	0.099	0.986	2.7	48.6
Crustacean	3.762	0.055	0.005	0.005	.281	1.937	0.088	0.094	1.118	0.49	8.8
Diptera	4.477	0.06	−0.007	0.005	.18	0.839	0.049	0.117	1.166	−0.65	−11.7
Ephemeroptera	3.752	0.056	−0.013	0.005	<.01	1.691	0.075	0.099	1.279	−1.08	−19.5
Hemiptera	−2.057	0.075	−0.012	0.006	.056	2.929	0.112	0.108	1.639	−1	−18
Megaloptera	−2.632	0.118	0.001	0.011	.917	2.636	0.121	0.215	1.548	0.1	1.9
Mollusc	3.536	0.073	0.007	0.007	.311	1.777	0.086	0.138	1.156	0.66	11.9
Odonata	−2.056	0.18	0.009	0.016	.574	2.992	0.089	0.351	1.336	0.93	16.8
Plecoptera	−1.218	0.104	0.052	0.008	<.001	4.496	0.092	0.157	1.331	7.89	142.1
Trichoptera	3.128	0.052	0.024	0.004	<.001	1.934	0.084	0.088	1.016	2.82	50.8
Turbellaria	−0.002	0.082	−0.043	0.007	<.001	2.462	0.148	0.143	1.517	−2.88	−51.8

**FIGURE 3:**
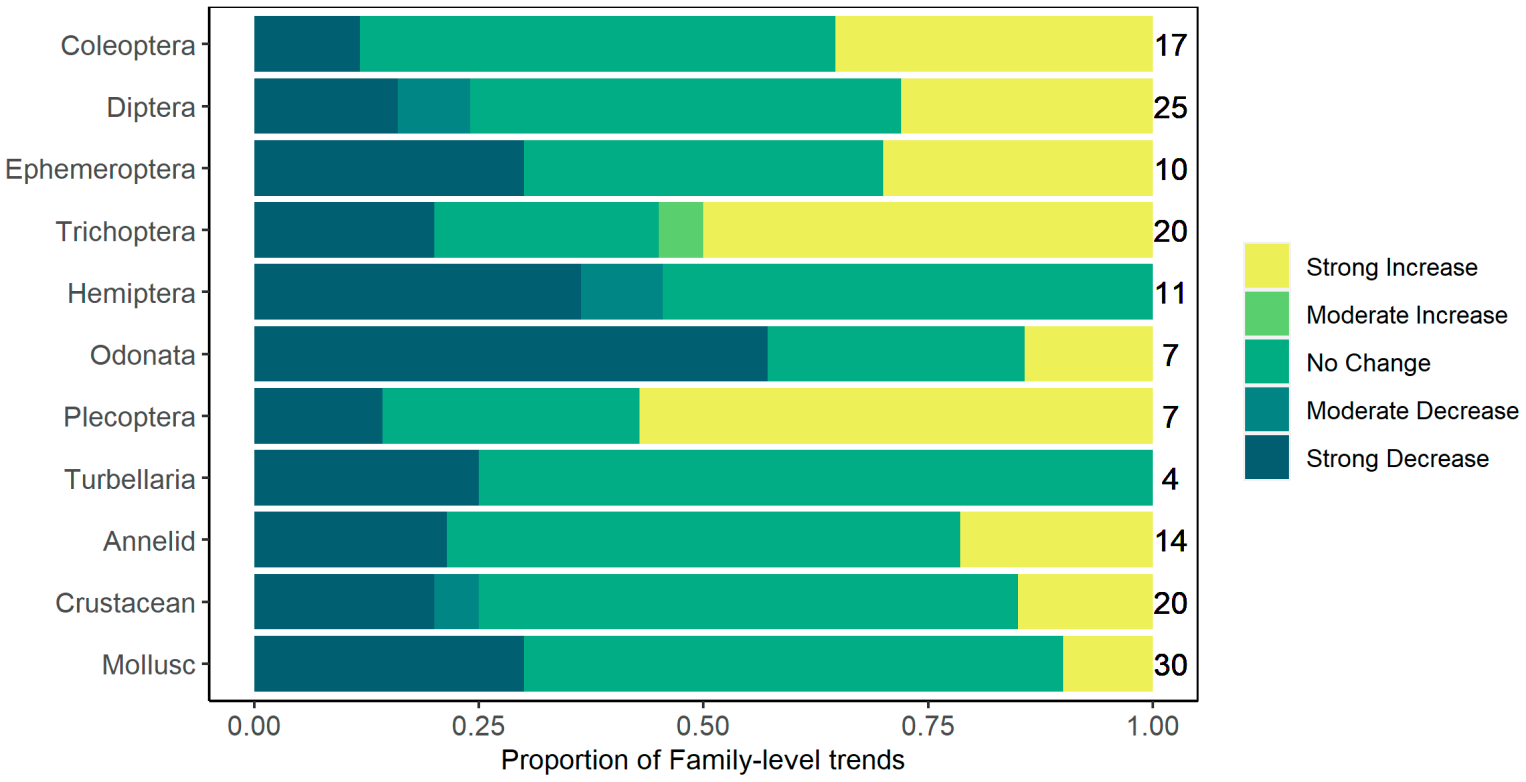
Proportion of family‐level trends analysed that show: (a) strong increases (where the annual growth rate ≥2.81%, leading to a doubling of abundance over 25 years); (b) moderate increases (where the annual growth rate is between 1.16% and 2.81%); (c) No change (where trends were insignificant—All trends with growth rates between −1.14% and 1.16% were insignificant), (d) moderate decreases (where the annual growth rate is between −2.73% and −1.14%); and e) strong decreases (where the annual growth rate ≤−2.73%, representing at least a halving of abundance over 25 years). *N* = 67,753 site‐sample combinations. Family trends are represented as proportion of families we were able to analyse (given data limitations) within wider taxonomic groups, with the total number of families analysed given on the right of each bar.

Data aggregated to families also showed variable trends (Figure 3, Table S3). Almost half of all analysed families (82 of 166) show ‘no change’ with no significant linear abundance trends over time, including families for which significant trends were found at higher taxonomic levels (e.g. Turbellaria and annelids; Table 2). Of the significant family trends, an approximately even number of families were found to increase in abundance (41 families) and decrease in abundance (43 families; Table S3).

#### 3.1.2 Trophic group abundance

Both herbivore and carnivore abundances increased, by an estimated 14.8% and 19% respectively over 18 years. Over the same time period, decomposers decreased in abundance by approximately 21.7% (Figure 2a, Table 2).

### 3.2 Trends by river typology

Models that allowed for trends to vary across river typologies identified significant typological variation in trends (Table S2).

#### 3.2.1 Wider taxonomic group abundance

Abundance trends for wider taxonomic groups across river typologies in some cases diverged from their national averages (Figure 5). For example, Ephemeroptera decreased in calcareous and siliceous rivers at low altitude (most sites) but were stable across other typologies and increased in calcareous rivers at higher altitudes by 29% (Table S2). At a national level, Odonata showed stable (non‐significant) trends, but Odonata trends increased significantly in calcareous rivers at high altitudes, with an estimated abundance increase of 123% (Table S2). In contrast, other groups showed little divergence from the overall national abundance trend when river typology was taken into account; for example, annelids had no positive trends across river typologies, and only organic rivers and low altitudes were found to have stable, non‐significant trends for this group. Turbellaria, the invertebrate group with highest overall decline at the national scale, were found to be significantly increasing over time in this same type of river (organic rivers at low altitude; an increase of 550%. Table S2). Estimates for all taxonomic groups and river typologies are shown in Table S2.

#### 3.2.2 Trophic group abundance

**FIGURE 4:**
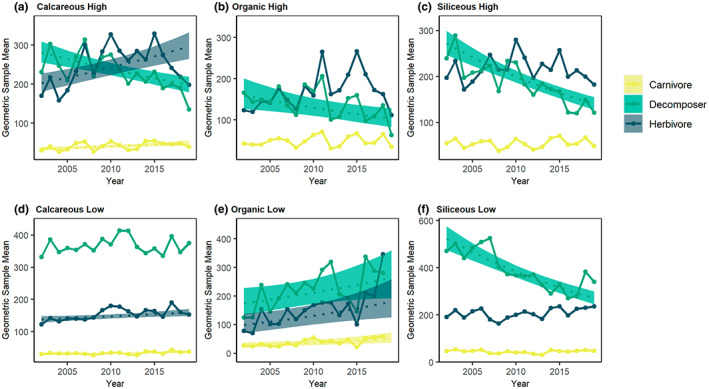
Abundance of river macroinvertebrates from 2002 to 2019 for carnivores, herbivores and decomposers in samples taken from rivers of different river typology category. Abundance is presented as the geometric mean number of individuals per 3‐min kick sample shown with a solid line. Dashed lines show model predictions based on the raw data for groups where the effect of ‘year’ on abundance was significant (*p* ≤ .05), with shaded envelopes indicating 95% confidence intervals, for the following typology categories: (a) calcareous high (*n* = 7233), (b) organic high (*n* = 1099), (c) siliceous high (*n* = 8032), (d) calcareous low (*n* = 44,566), (e) organic low (*n* = 599) and (f) siliceous low (*n* = 6228), where *n* = number of site‐sample combinations.

Abundance trends in trophic groups also varied among river typologies (Figure 4, and Tables S1 and S2). For example, herbivorous macroinvertebrates had no significant trends across half of our river typologies, only increasing in abundance in calcareous rivers and organic rivers at low altitude (Table S2; Figure 4). Although trends for herbivore abundance were significant and positive in calcareous rivers at high altitude over the long term, the geometric mean abundance progressively decreases over the last 4 years of data collection (years 2015–2019; Figure 4a). This pattern also exists for herbivores in organic and siliceous rivers at high altitudes, which had no significant trend over the long term (Figure 4b,c). Figure S5 shows the results of GAMMs, including herbivores in the top right panel; these supplementary results show a non‐linear trend that captures this short‐term decline towards the end of the time series.

**FIGURE 5:**
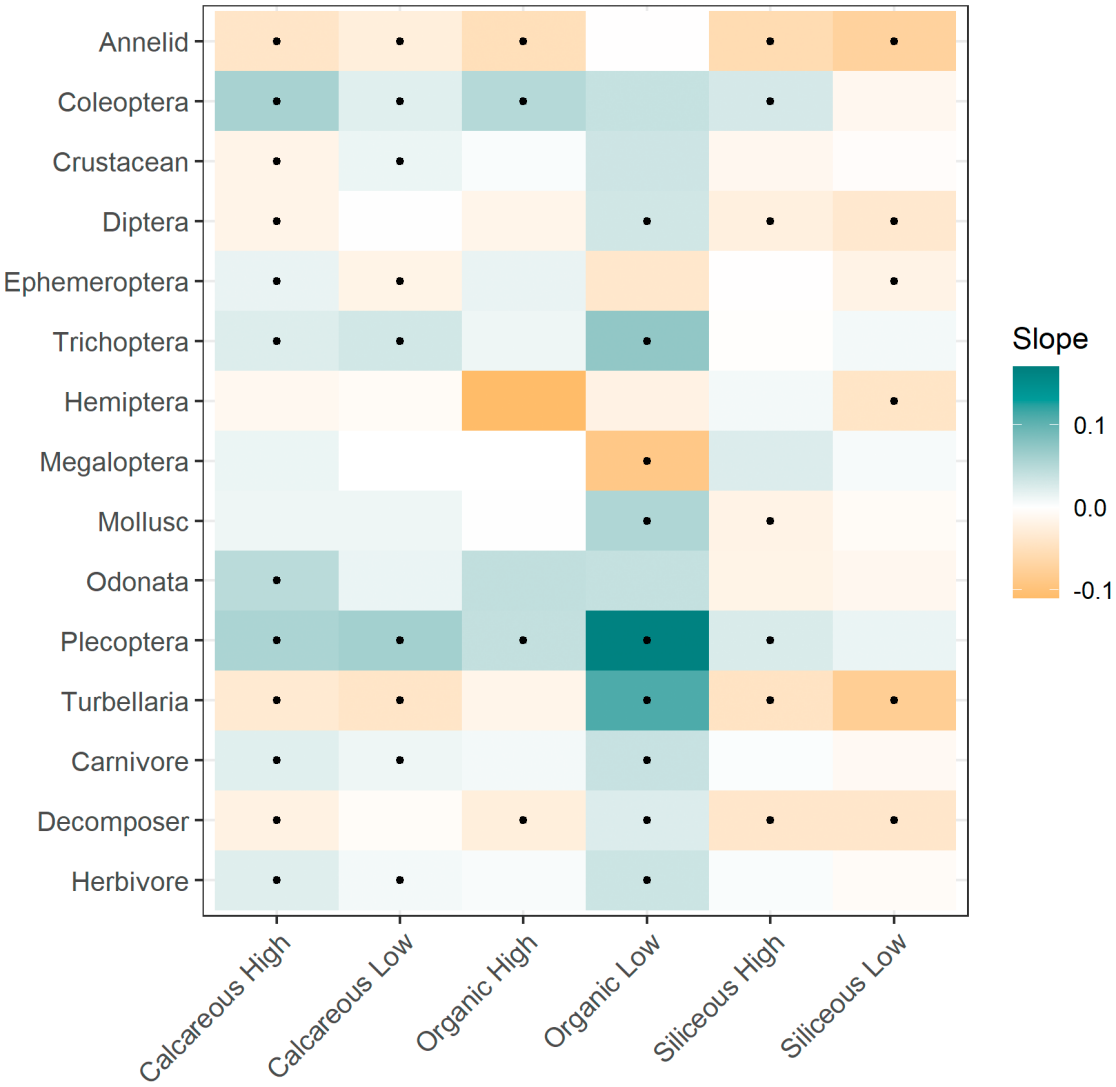
Trend slopes (*β* values) for the models testing the interaction between year and river typology category (model 2), for each broader taxonomic and trophic group. Significant trends (*p* ≤ .05) are represented by a black dot. The number of sample‐site combinations for each river typology is as follows; calcareous high: *n* = 7233, calcareous low: *n* = 44,566, organic high: *n* = 1099, organic low: *n* = 599, siliceous high: *n* = 8032 and siliceous low: *n* = 6228.

Carnivores increased on a national scale, but again their trends were found to be stable in siliceous rivers and organic rivers at high altitude, with only calcareous rivers and organic rivers at low altitude showing significant trends. Finally, we found decomposers to be declining across all river typologies apart from low altitude calcareous rivers (where there were no significant trends) and organic rivers and low altitude, where decomposer macroinvertebrates were found to be significantly increasing in abundance (Table S2; Figure 4).

## 4 DISCUSSION

Our study capitalizes on a unique long‐term abundance data to describe and compare changes in abundance of freshwater macroinvertebrates at a national scale and across different types of river. We report a range of positive, negative and stable trends in macroinvertebrate abundance over time, with the direction of these trends depending on taxonomic and trophic groupings and varying with river typology. Stable trends have been reported in recent meta‐analyses of freshwater invertebrates across continental scales and in the United States (Crossley et al., 2020; van Klink et al., 2020); however, these studies did not quantify spatial and taxonomic heterogeneity in abundance patterns, as we do here. Although our results find that abundance trends are inherently complex within freshwater macroinvertebrate communities, there may be important consequences for changes in ecosystem function provision through a shift in the abundance within different trophic levels. Our results have implications for management of freshwater ecosystems, highlighting particular river types that are most susceptible to invertebrate abundance declines.

### 4.1 Heterogeneity of trends

Although many indicators suggest we are losing biodiversity around the globe (Johnson et al., 2017; Wagner, 2020), caution is required when inferring widespread losses from higher level groupings (e.g. by Order or trophic level) (Leung et al., 2020). While there is evidence of decline in many terrestrial invertebrates (Wagner, 2020; Wagner et al., 2021), here we show stable and increasing trends among several freshwater macroinvertebrate taxa in England. We also show that although freshwater ecosystems in England do not appear to be suffering general macroinvertebrate declines at the national level, the pattern of change across taxonomic groups and across space is more complex and variable than simplistic summary statements allow for. We must consider this spatial and taxonomic variation as an important part of the conversation around the state of invertebrate populations and biodiversity change (Cardinale et al., 2018; Chase et al., 2018, 2019). This complexity is likely to be representative of heterogeneity in multiple environmental stressors, which is at risk of being overlooked if different ecological scales are not considered (Simmons et al., 2021).

Our work highlights the value of long‐term abundance data collected through standardized monitoring schemes to reveal complexity, and new patterns of heterogeneity not observed in previous studies of freshwater ecosystems using presence/absence and diversity metrics. Of the family‐level trends that we were able to quantify, almost half of all trends are non‐significant; coupled with the positive trends, we found no evidence that most families are declining in abundance. In addition, we observed variation in family‐level trends within wider taxonomic groups—showing that although total abundance may not be changing significantly in some groups, there could be significant turnover in biodiversity within groups as some families increase and some decrease in abundance over time. One extreme example, Odonata, showed no significant trend in total abundance overall, but most families showed strong declines in abundance. Their declining trends were masked when analysed together because the most abundant odonatan family, Libellulidae, has had largely stable population sizes since 2002, and a few other families showed increases. Conversely, we found families with contrasting trends in groups for which overall estimates showed significant declines or increases including Ephemeroptera, Trichoptera, Plecoptera, annelids and Turbellaria.

### 4.2 Drivers of freshwater macroinvertebrate abundance change

Most comparable studies have identified water quality improvement in England over the last few decades as an explanatory factor for macroinvertebrate biodiversity trends (Environment Agency, 2021; Outhwaite et al., 2020; Vaughan & Gotelli, 2019; Vaughan & Ormerod, 2012b, 2014). We found annelid worms, which are often associated with poor water quality due to their high tolerance to organic pollution (Armitage et al., 1983), have declined significantly across all‐but‐one of the river typology categories—organic lowland rivers. By contrast, we found other macroinvertebrate groups generally associated with better water quality due to higher sensitivity, such as some families of Plecoptera and Trichoptera, to have generally increased (Table S2). Within groups and orders of macroinvertebrates, different families can vary in their sensitivity to environmental drivers such as organic and chemical pollution (Hellawell, 1986). For example, for Trichoptera abundance, several more pollution‐tolerant families, such as Hydropsychidae and Hydroptilidae have not changed in abundance over time (Table S3), and several more sensitive taxa such as Goeridae and Odontoceridae have increased. However, the state of water quality improvement has halted and even reversed in the last 4 years in England; this warrants further investigation into how these recent changes in water quality may affect abundance and other indicators for macroinvertebrates going forward (Environment Agency, 2020b).

On the other hand, Ephemeroptera, also generally linked to high water quality, significantly decreased in abundance in our national‐scale analysis (Figure 2). Despite this our family‐level analysis shows that a number of sensitive families which score higher for water quality indication within Ephemeroptera are increasing, such as Ephemeridae, Siphlonuridae and Heptageniidae, whereas families which are less sensitive to pollution such as Baetidae were either stable or in decline. Beyond water pollution, other drivers of change such as light pollution can disproportionately affect taxa such as Ephemeroptera, and although we do not test for environmental drivers, the presence of a wide range of stressors such as these may contribute to the different patterns seen across broader groups of taxa (Kriska et al., 1998).

Conditions and impacts affecting different types of rivers could also drive variation in trends. Broadly speaking, calcareous rivers tend to have more positive trends across taxonomic and trophic groups than siliceous rivers, which appear to have largely negative trends (with some exceptions in both cases). The calcareous rivers in England, which include limestone rivers and rarer chalk streams and rivers, are typically fed more by groundwater than surface waters in England and, as a result, tend to be subject to different river conditions to siliceous and organic rivers (Berrie, 1992). Calcareous rivers can provide a more stable environment than surface water‐fed siliceous rivers for freshwater species. This is because the former are generally less susceptible to fluctuations in flows, flood events and droughts, and the resulting ‘wash out’, high velocity, temperature and dissolved oxygen fluctuations, along with pollutant concentrations, that come with flow changes (Eveleens et al., 2019; Ledger & Milner, 2015; Mosley, 2015; Piniewski et al., 2017). It is possible that rivers with higher base flows are providing a more stable environment to support richer invertebrate communities benefiting from the wide scale water quality improvements documented elsewhere (Vaughan & Ormerod, 2012b). However, Whelan et al. (2022), shows that changes in water quality in the United Kingdom are complex; although phosphate loading and acidification appear to have recovered somewhat, catchments with intensive agriculture are likely to be fairing worse than pre‐1960 levels of water quality (Whelan et al., 2022).

Organic sites—in areas dominated by peatland—generally have the strongest increases in macroinvertebrates, especially in lowland rivers. There are much fewer organic river sites in England than siliceous and calcareous rivers, and our trends are likely inherently susceptible to spatial autocorrelation due to the aggregation of sites in areas dominated by particular sediment types. For example, there is an aggregation of lowland organic sites in Anglia, which lie in the Fens (Figure 1). We note that Diptera are either significantly decreasing or have no significant trend in other sites; this is likely driven by the families that tend to be found in high abundance but that we found to be strongly declining, such as Chrinomidae and Simuliidae. However, Diptera increase significantly within organic lowland sites; if driven by Chirnomid and Simuliid abundance change this would not support our hypothesis that these particular sites are subject to significant increases in water quality.

### 4.3 Ecosystem functioning

Ecosystem functions and services are often disproportionately driven by the abundance of common species (Larsen et al., 2018; Winfree et al., 2015), and so monitoring population and group‐level changes of macroinvertebrate abundance—instead of occurrence, which is more sensitive to rare and vulnerable species—can ultimately contribute to a more detailed understanding of ecosystem function (Greenwell et al., 2019). Freshwater macroinvertebrates support a number of different ecosystem functions and services (Macadam & Stockan, 2015), but namely they constitute the bulk of the diet of many fish, bird and bat species, including some rare and protected species in England such as the Daubenton's bat (*Myotis daubentonii*) and the Eurasian Dipper (*Cinclus cinclus*), whose diet is largely made up of Trichoptera. Identifying long‐term declines in the abundance of families and wider taxonomic groups of freshwater macroinvertebrates can inform on the availability of food sources for these higher trophic levels.

Trophic level changes such as those we show here may have consequences for regulatory ecosystem services associated with freshwater systems such as water self‐purification processes (Ostroumov, 2017). We suggest that an increasing abundance of herbivorous and declining decomposer abundance represents a trophic level shift within macroinvertebrate communities, although they are still largely dominated by decomposers. Herbivorous invertebrate increases are being driven by a number of increasing families within Coleoptera, Trichoptera and Plecoptera, while carnivorous abundance increases reflect increases in invertebrate‐feeding Coleoptera, crustaceans, Odonata and Megaloptera (in some river typologies). Decomposer abundance decline reflects changes in some abundant dipteran and annelid families. Decomposer declines may be driven by lower abundances in pollution‐tolerant groups such as oligochaetes and flow regime change and sediment pollution, but regardless of the drivers these declines could result in stagnation of the self‐purification process through leaf‐litter breakdown and removal, a vital process in freshwater ecosystem functions (Mustonen et al., 2016). Further analyses would be needed to investigate the potential repercussions of the trophic level changes we highlight in this study.

### 4.4 Limitations and caveats

Although we discuss the potential consequences of our findings for ecosystem functions and services, future studies using biomass and dietary preference data could give a more nuanced picture of the functional consequences of temporal invertebrate community change (Lu et al., 2016). Using biomass would provide a more accurate picture of the state of food and energy availability for predator species in freshwater ecosystems. Similarly, combining biomass data with other functional traits could reveal more about ecosystem functions such as decomposition, as organisms with larger biomass consume larger amounts of food. If, for example, decomposer declines are driven to a significant degree by Chironomids, which we found to be declining significantly over time, then hypothetically, increases in other decomposers of higher biomass could prevent or mitigate the loss of function. Biomass data and organic matter feeding/decomposition rates are not captured in this monitoring scheme but extending monitoring to consider a functional trait approach holds promise for future research.

Additionally, our method of calculating dietary preferences may have resulted in some taxa having greater influence over results, for example where the fuzzy‐coded data in Tachet et al. (2015) sum to greater values across dietary components, meaning we had a potentially reduced capacity to estimate the diet of some individuals which were not identified down to genus level, although we do not think this would have had much of an impact on our results due to our method of weighting by genus presence.

We emphasize the importance of long‐term data to evaluate biodiversity changes, but even analyses covering nearly two decades, such as the one analysed here, have limitations. We were not able to resolve species or genus‐level trends, which has limited our ability to understand the potential reasons for increases and declines identified in our dataset. Although we discuss family‐level trends in the context of water quality changes (due to different families varying in response to water quality improvement and pollution), within families there is also variation among species in their sensitivity to water quality metrics, or their ‘saprobic index’, which we were not able to capture in this analysis (Metcalfe, 1989). Nor were we able to calculate absolute abundance change earlier than 2002, due to the limitations of the dataset explained earlier. Although our study presents a range of trends from declines to stable and increasing abundance of freshwater macroinvertebrates since 2002, current population sizes may actually be much lower in English rivers than 50 or 100 years ago.

Finally, the dynamics of invertebrate trends are difficult to capture and model over the long term due to high interannual variation that is inherent across these taxa (Baranov et al., 2020; Cauvy‐Fraunié et al., 2020); this appears to also be the case with our data, shown in figures 2 and 3. We have chosen to model long‐term abundance change of macroinvertebrates using hierarchical linear modelling, and while this approach allows us to provide our best estimate of how abundance has changed on average since 2002, the models presented do not capture changes from 1 year to the next, nor explain occasional short‐term non‐linear patterns in geometric means. For example, some patterns that appear to buck the linear trend—such as herbivore abundance in the latter years of the dataset—may well be better represented by non‐linear modelling such as using generalized additive models; for this reason, we provide additional models in the Supporting information that represent these short‐term patterns. Other important questions about macroinvertebrate abundance change in the United Kingdom and more widely remain, such as the stability and resilience of these communities over time under fluctuating environmental extremes, which are increasing under climate and land use change pressures (Fried‐Petersen et al., 2020; Jourdan et al., 2018).

### 4.5 Implications and recommendations

Our work has important implications for policy in the United Kingdom and beyond. In the wake of the UK's exit from the European Union, new policies and targets have been created to replace EU biodiversity and environmental policy, for example, the UK Government's 25 Year Environment Plan and the Environment Act (2021). This legislation has triggered new targets in England to halt the decline of species abundance by 2030 and increase abundance by 10% by 2042 (although these are currently subject to change). Although we were unable to identify species‐level trends using this dataset, our higher taxonomic level and trophic abundance trends highlight particular groups, such as Ephemeroptera, that have fared worse than other groups of macroinvertebrates, warranting further investigation into invertebrate abundance declines in England. Our analysis also highlights particular river types where macroinvertebrates have declined at higher rates, in particular, siliceous rivers, which are less likely to be resilient to ex situ environmental pressures, such as pollution from agricultural run‐off. We suggest this could help direct future management and conservation interventions towards particular river types whose macroinvertebrate communities are more vulnerable.

In view of our use of a Water Framework Directive‐based typological approach to river characterization in this study, we suggest that our results could be used in the future to compare across river systems across Europe, where there are similar macroinvertebrate sampling procedures and typological classifications of rivers. We hope this approach could be used to investigate trends and direct further research and management on a European‐wide scale for different types of river typologies based on patterns of abundance change across macroinvertebrate communities. Following the UK's exit from the European Union, regardless of future legislation following the EU Water Framework Directive, we recommend that future monitoring of macroinvertebrate communities in England under the Environment Agency continue to use the same sampling and monitoring protocol to make new data on abundance and biodiversity comparable to past data, as well as to the rest of Europe.

In conclusion, extensive monitoring schemes and detailed analyses that explore taxonomic, functional and spatial nuances are necessary if we are to better understand the extent of biodiversity change around the world. Further studies are needed to predict how the provision and resilience of key ecosystem functions provided by freshwater communities are affected by abundance changes within individual invertebrate taxa and for specific catchments, and to identify key anthropogenic drivers to aid targeted ecosystem management.

## CONFLICT OF INTEREST

The authors declare no competing interests.

## Supporting information


Appendix S1

Appendix S2

## Data Availability

The data that support the findings of this study are available in Dryad at http://doi.org/10.5061/dryad.v9s4mw70n. These data were derived from the following resources available in the public domain: https://environment.data.gov.uk/ecology‐fish/. The R scripts used to produce the trends for this study can be found at https://github.com/katpow/river_macroinvertebrate_trends.
