# Supplementary material for: Abundance trends for river macroinvertebrates vary across taxa, trophic group and river typology
Source: Glob Chang Biol. 2022 Dec 15;29(5):1282–95. doi: 10.1111/gcb.16549 (PMC10107317; doi:10.1111/gcb.16549)
Supplement: Supplementary file 1 — Appendix S1 [file GCB-29-1282-s001.docx]

**Supplementary Figures**


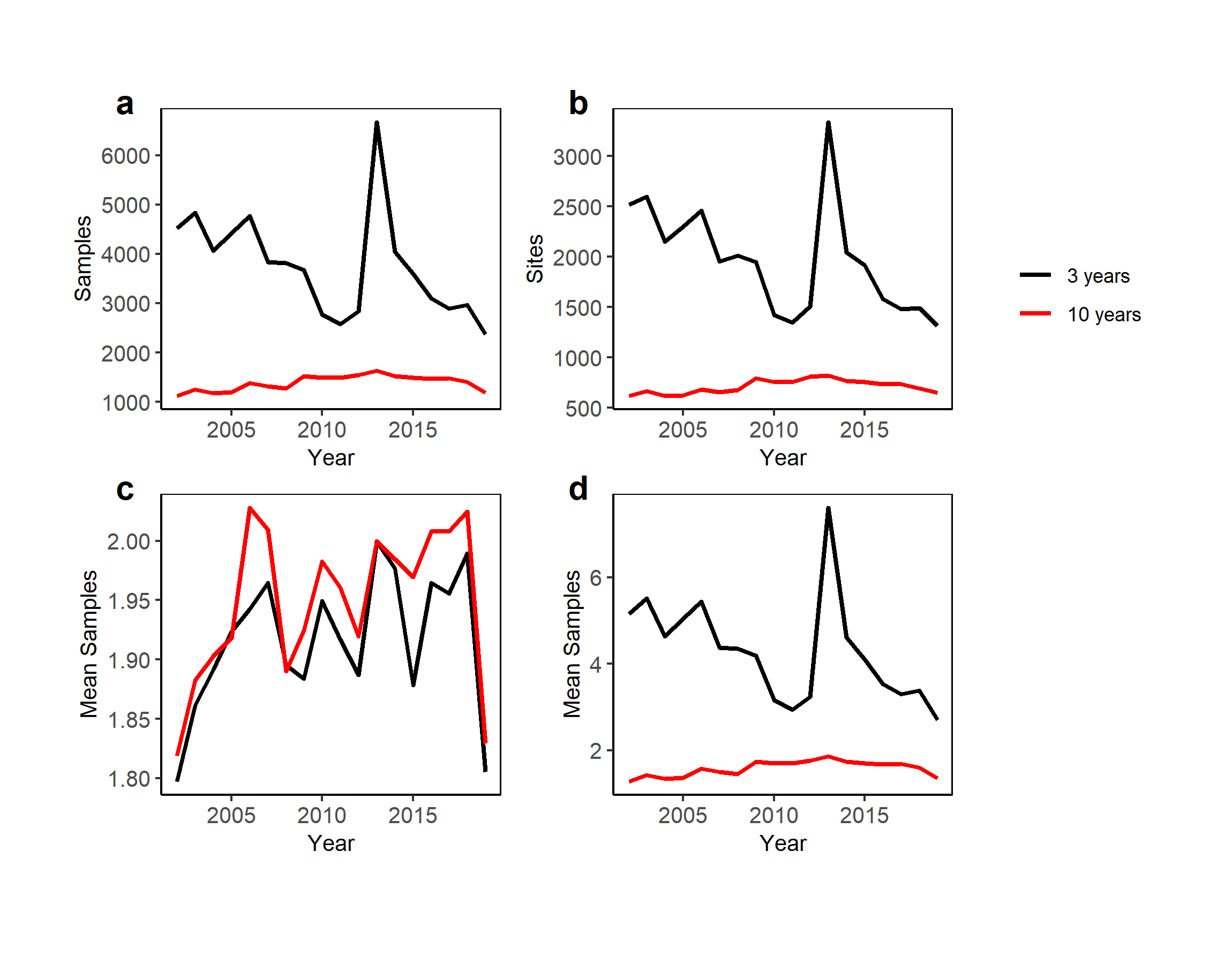


**Fig S1.** Comparison of sampling effort between two potential datasets to use for mixed effects models – one including only sites sampled in both spring and autumn for at least three years between 2002 and 2019 (black) and the other including only sites sampled in both spring and autumn for at least 10 years between 2002 and 2019 (red). Panel a shows the total number of samples taken each year; panel b shows the total number of sites included in the analysis each year; panel c. shows the mean number of samples taken per site included in the analysis each year, and d. shows the mean number of samples taken per total number of sites in the dataset each year.


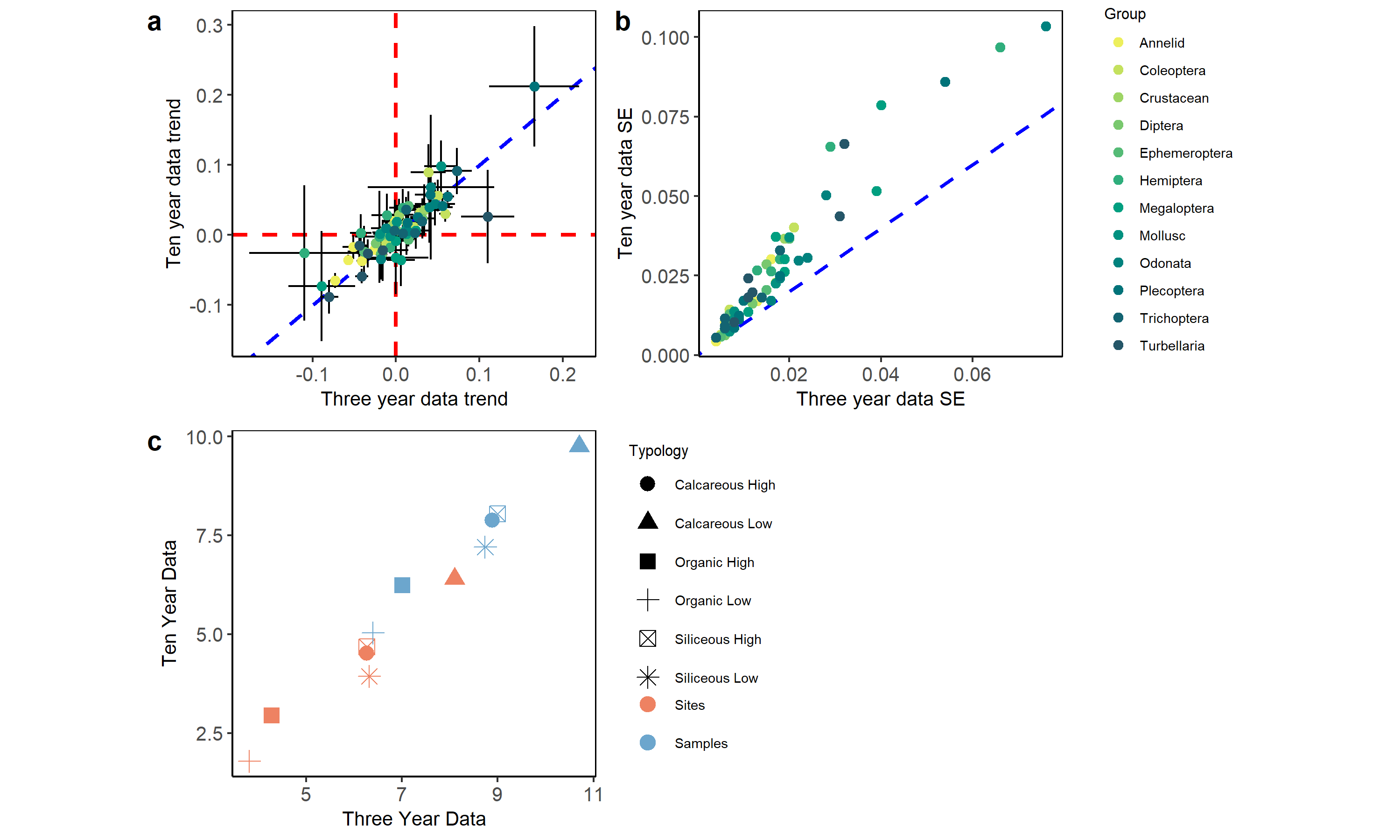


**Fig S2.** Comparison between two potential datasets to use for mixed effects models – one including only sites sampled in both spring and autumn for at least three years between 2002 and 2019 and the other including only sites sampled in both spring and autumn for at least 10 years between 2002 and 2019. Panel a: comparison of trends extracted from mixed effect model outputs including standard errors (Pearson’s correlation coefficient=0.85); panel b: comparison of the standard error for trends extracted from mixed effect model outputs (Pearson’s correlation coefficient=0.97); panel c. comparison of the number of sites and samples used in analyses (presented as log_e_ values due to scale differences).


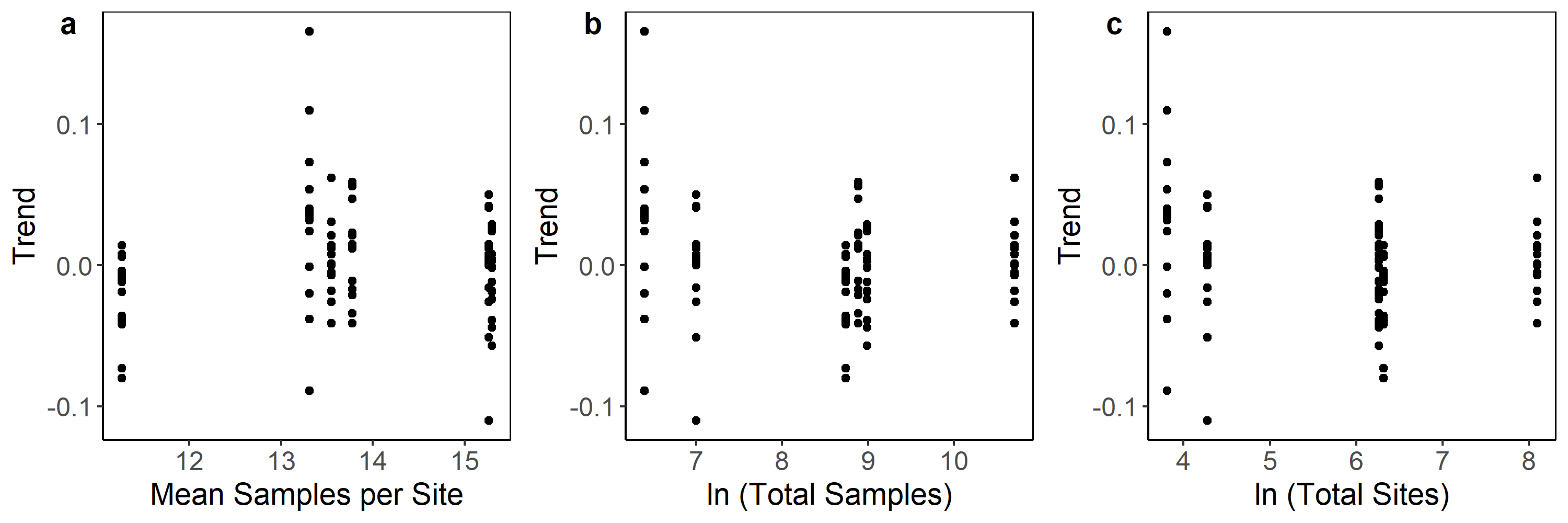


**Fig S3.** The relationship between a. the mean number of samples taken per site within each river typology; b. the total number of samples taken within river typologies and c. the total number of sites within each river typology; and the group-level trends extracted from final mixed effect model outputs (lm; β= -0.00495, s.e.=0.003, d.f.=88; p>0.05)


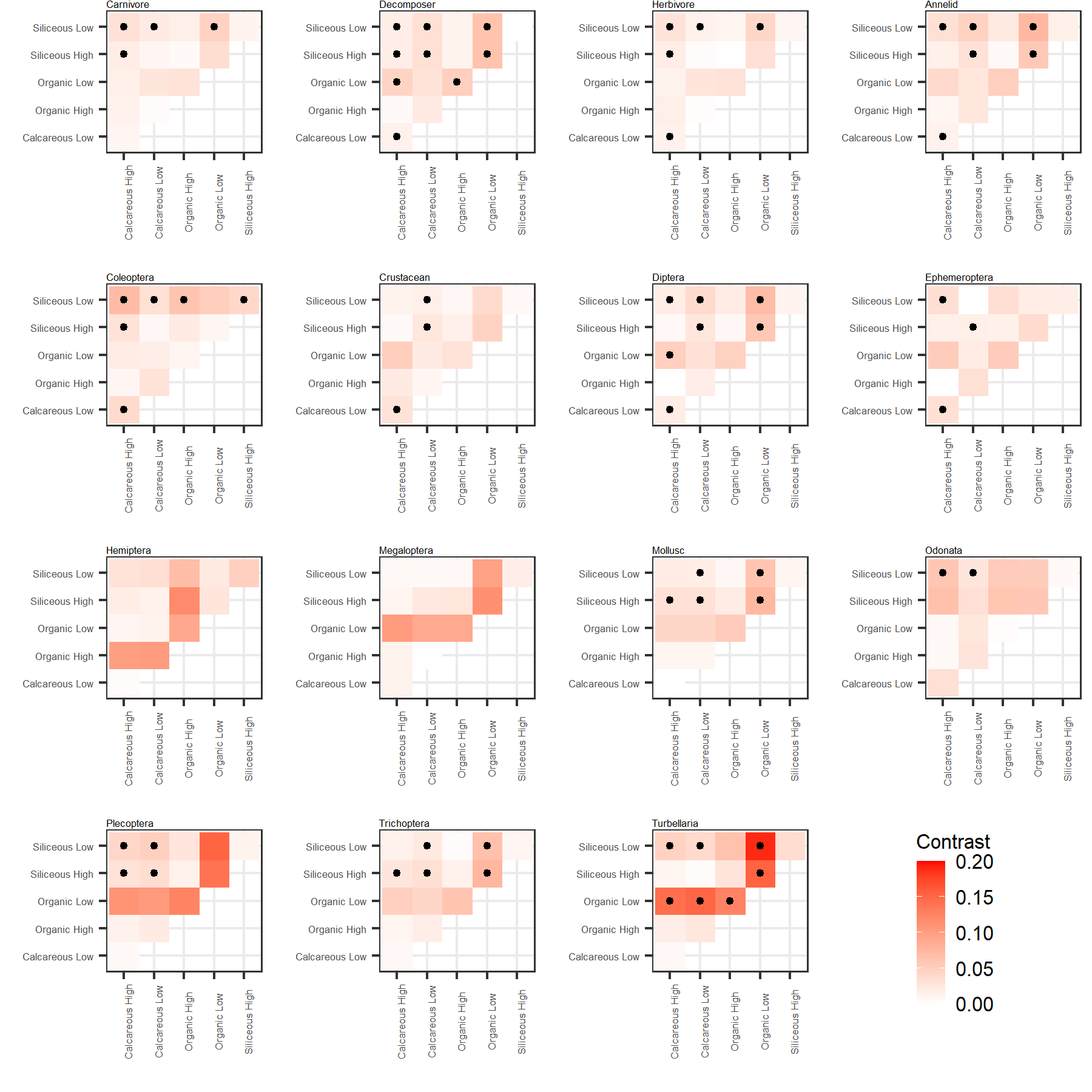


**Fig S4.** The contrast between different river typology trends in macroinvertebrate abundance change, for each trophic and taxonomic group, according to Tukey pairwise comparison tests. Darker colours show higher contrasts between river typologies, meaning there is a larger difference between their trends. Black dots show where the contrasts between trends are significant at the p≤0.05 level.


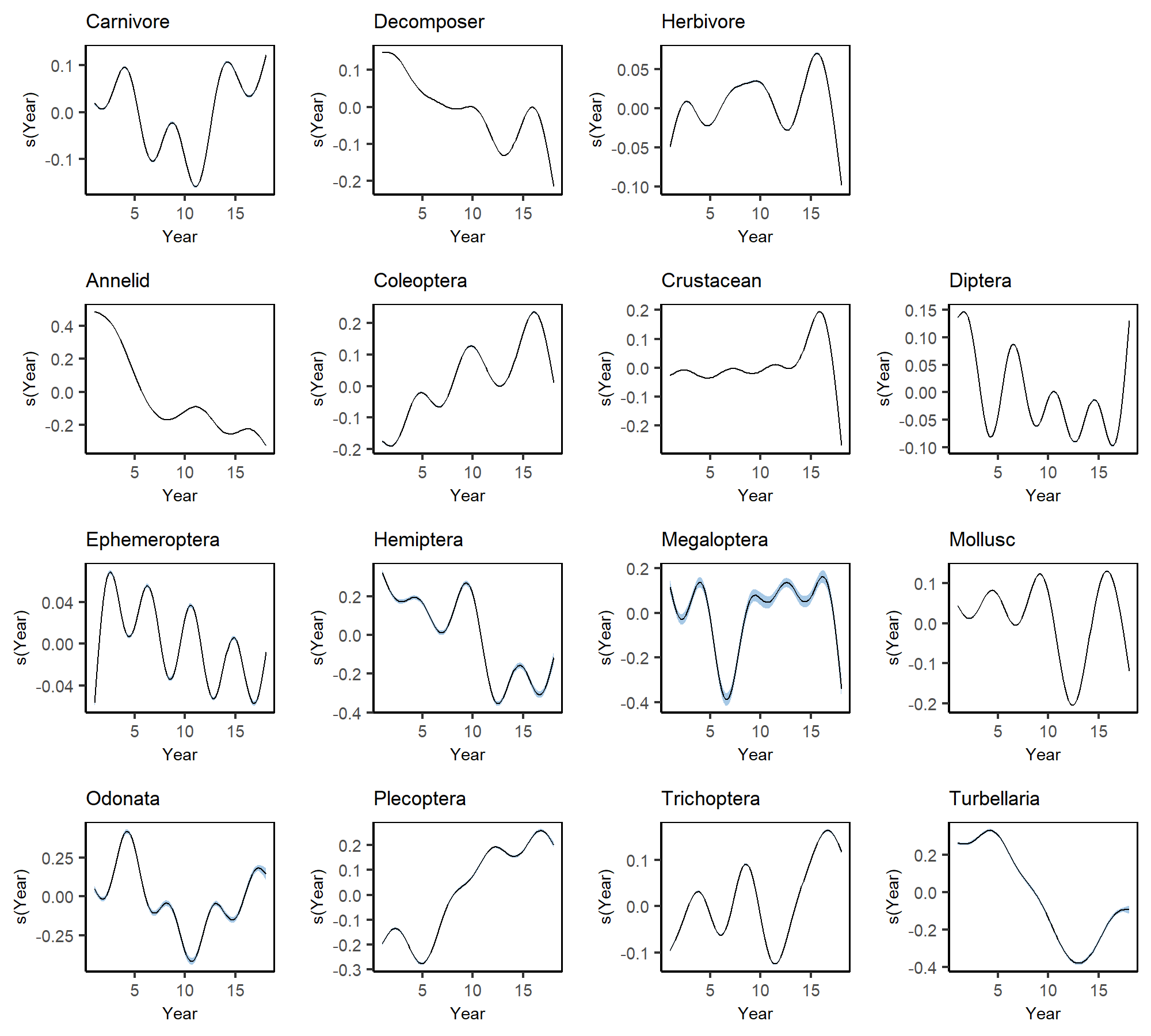


**Fig S5.** Plotted smooths of ‘Year’ fixed effects from GAMM trials, fitting site and observations as smoothed random effects in models. ‘Year’ is presented as integers 1-18 for the years 2002-2019. Approximate significance of smoothed terms can be found in Table S5.


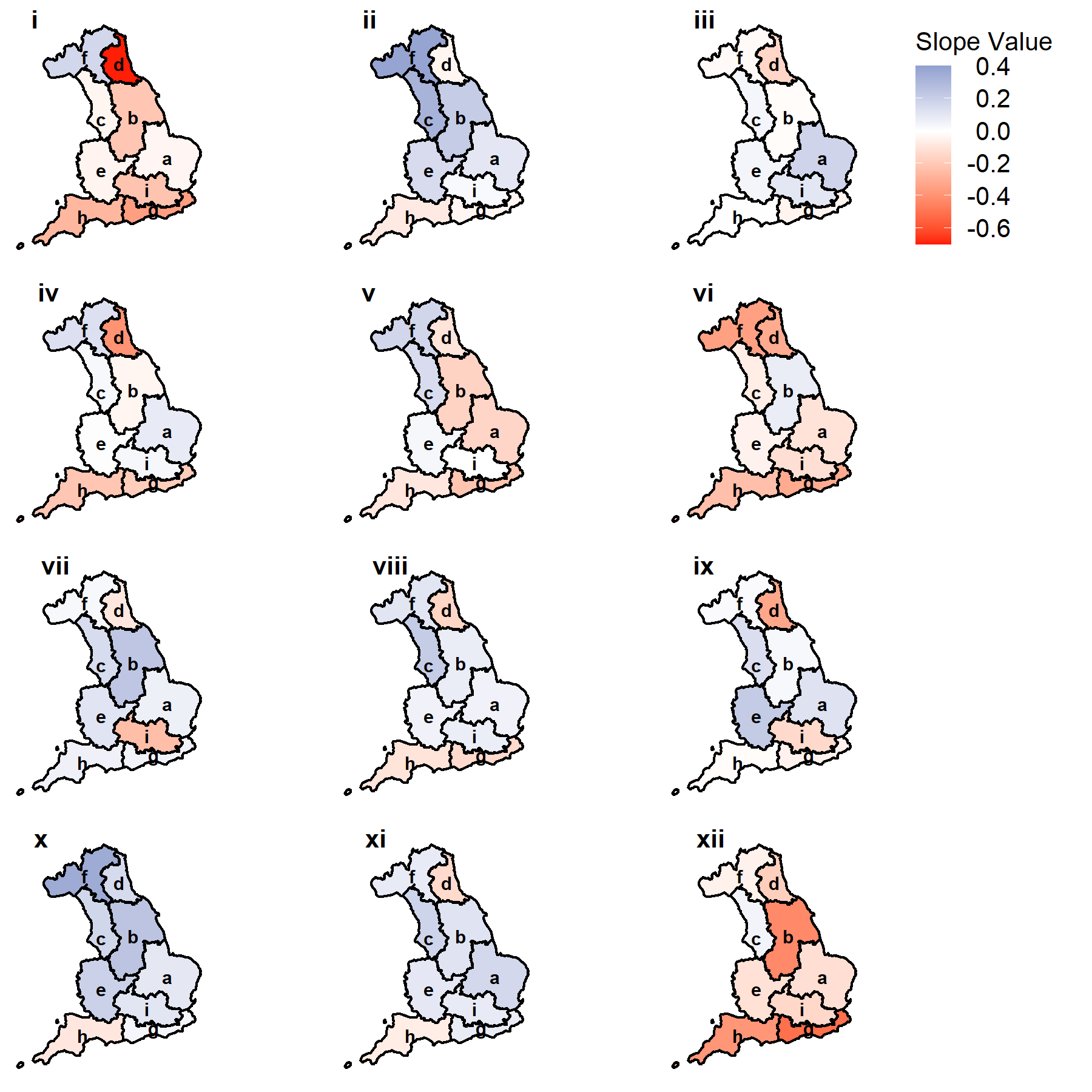


**Fig S6.** Map of English river basin district boundaries coloured by slope values extracted from models exploring spatial variation in trends, for i) annelids, ii) coleoptera, iii) crustaceans, iv) Diptera, v) Ephemeroptera, vi) Hemiptera, vii) Megaloptera, viii) molluscs, ix) Odonata, x) Plecoptera, xi) Trichoptera and xii) Turbellaria. Labels of river basins and the number of site:sample combinations are as follows; a) Anglian, n=12071; b) Humber; n=15396, c) North West, n=7519; d) Northumbria, n=3017; e) Severn, n=6727; f) Solway Tweed, 2101; g) South East, 4103; h) South West, n=7027, and i) Thames, n=8808. Map lines delineate study areas and do not necessarily depict accepted national boundaries.
