# Supplementary material for: Abundance trends for river macroinvertebrates vary across taxa, trophic group and river typology
Source: Glob Chang Biol. 2022 Dec 15;29(5):1282–95. doi: 10.1111/gcb.16549 (PMC10107317; doi:10.1111/gcb.16549)
Supplement: Supplementary file 2 — Appendix S2 [file GCB-29-1282-s002.docx]

**Abundance trends for river macroinvertebrates vary across taxa, trophic group and river typology (Supporting Information)**

Kathryn E. Powell*^1,^, Tom H. Oliver^2^, Tim Johns^3^, Manuela González-Suárez^2^, Judy England^3^, David B. Roy^1^

^1^ UK Centre for Ecology and Hydrology, Crowmarsh Gifford, Wallingford, Oxfordshire, OX10 8BB, UK

^2^ School of Biological Sciences, University of Reading, Reading, RG6 6AS, UK

^3^ Environment Agency, Thames Area, Howbery Park, Crowmarsh Gifford, Wallingford, Oxfordshire, OX10 8BD, UK


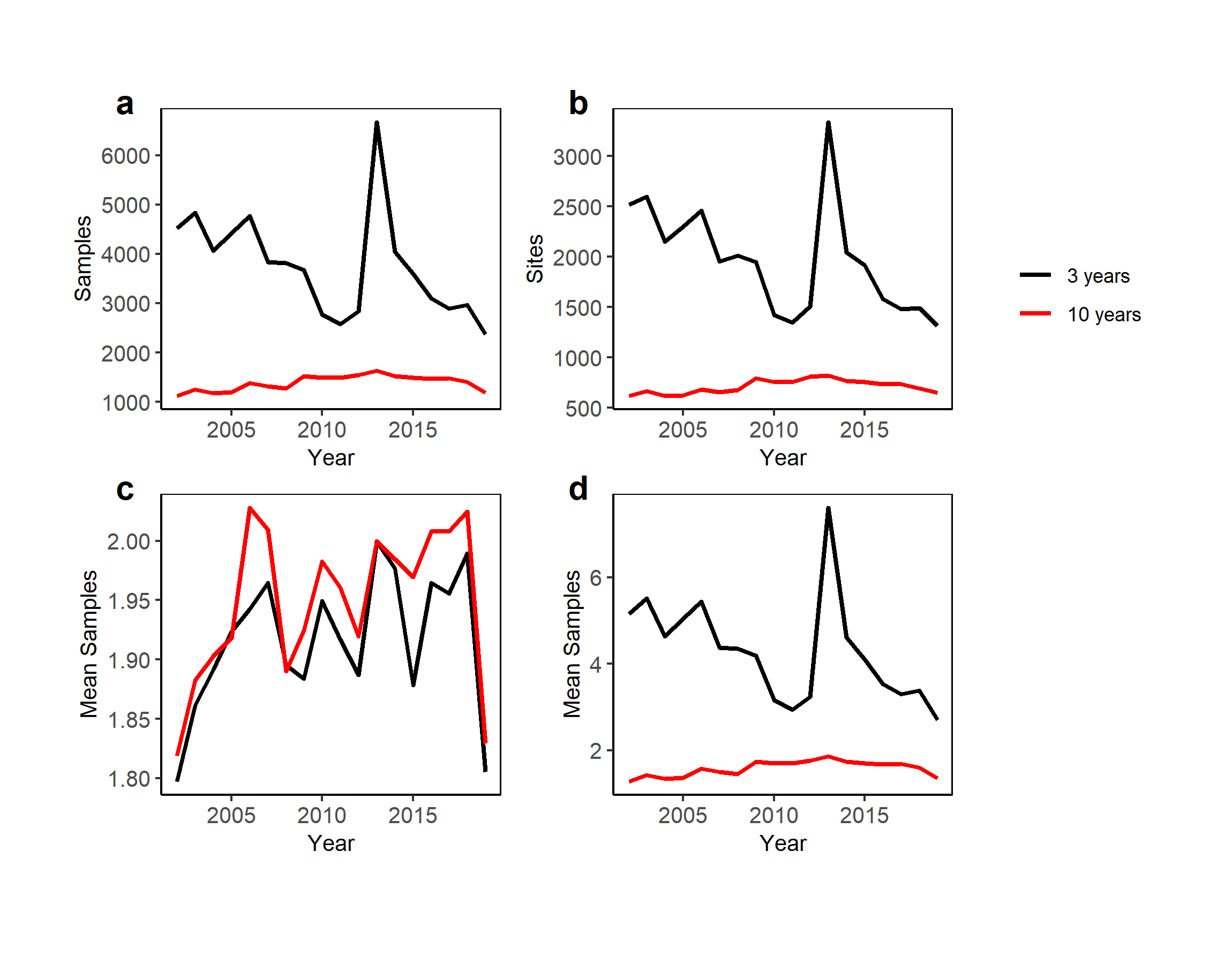
***Corresponding Author contact details:** katpow@ceh.ac.uk

**Fig S1.** Comparison of sampling effort between two potential datasets to use for mixed effects models – one including only sites sampled in both spring and autumn for at least three years between 2002 and 2019 (black) and the other including only sites sampled in both spring and autumn for at least 10 years between 2002 and 2019 (red). Panel a shows the total number of samples taken each year; panel b shows the total number of sites included in the analysis each year; panel c. shows the mean number of samples taken per site included in the analysis each year, and d. shows the mean number of samples taken per total number of sites in the dataset each year.


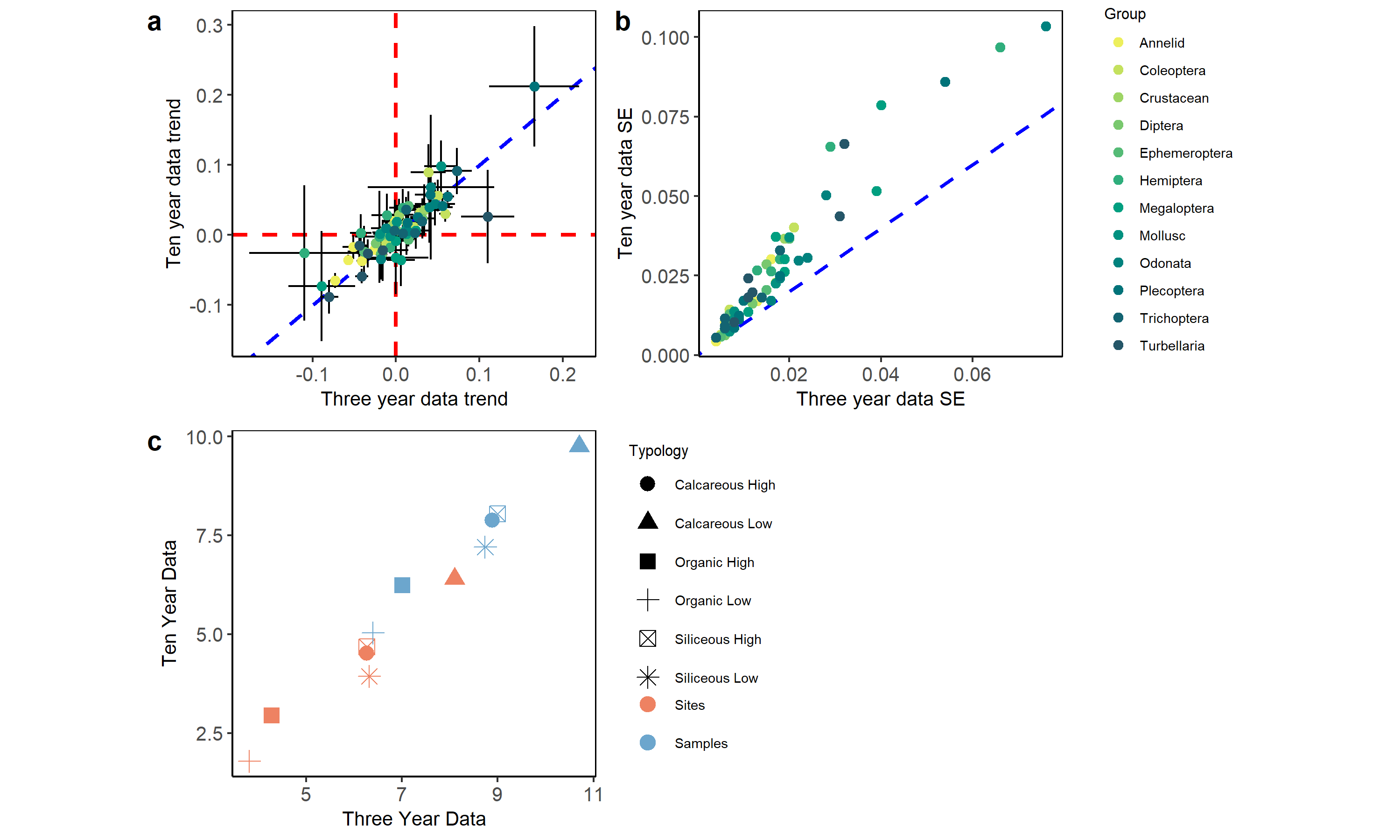


**Fig S2.** Comparison between two potential datasets to use for mixed effects models – one including only sites sampled in both spring and autumn for at least three years between 2002 and 2019 and the other including only sites sampled in both spring and autumn for at least 10 years between 2002 and 2019. Panel a: comparison of trends extracted from mixed effect model outputs including standard errors (Pearson’s correlation coefficient=0.85); panel b: comparison of the standard error for trends extracted from mixed effect model outputs (Pearson’s correlation coefficient=0.97); panel c. comparison of the number of sites and samples used in analyses (presented as log_e_ values due to scale differences).


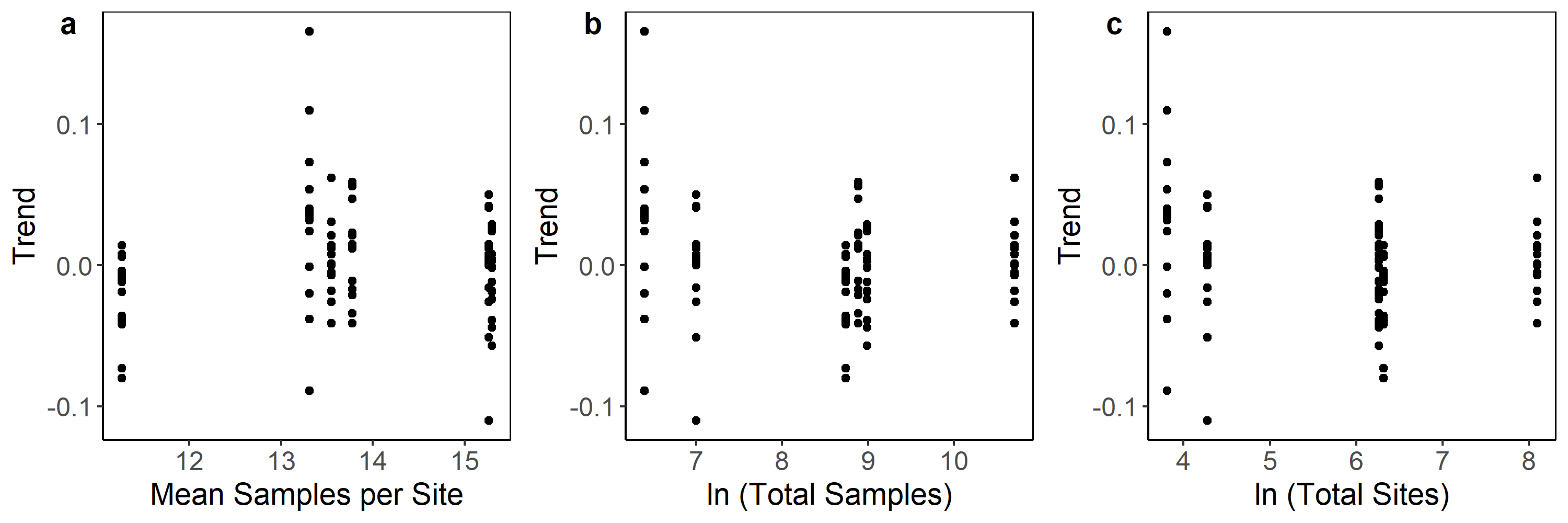


**Fig S3.** The relationship between a. the mean number of samples taken per site within each river typology; b. the total number of samples taken within river typologies and c. the total number of sites within each river typology; and the group-level trends extracted from final mixed effect model outputs (lm; β= -0.00495, s.e.=0.003, d.f.=88; p>0.05)


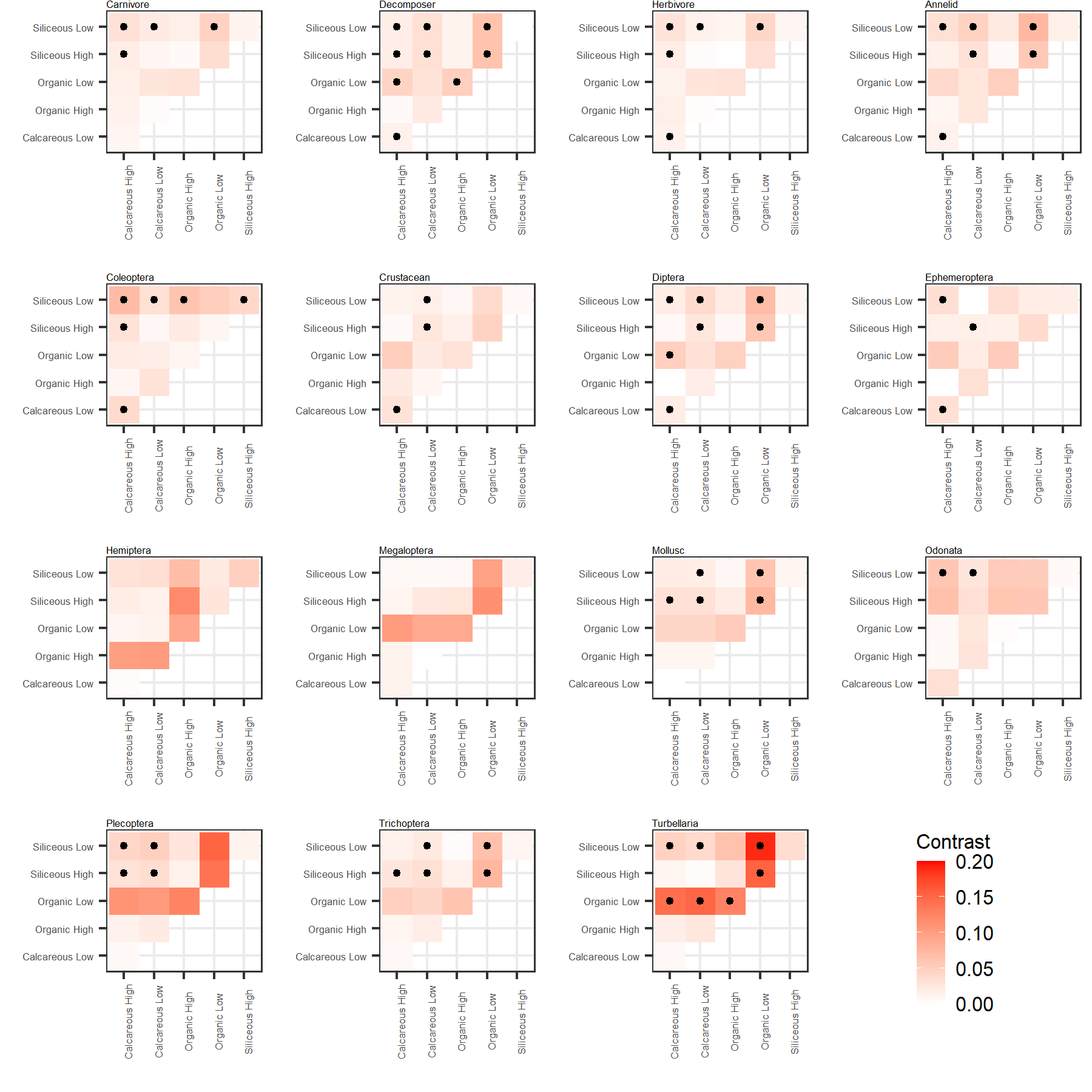


**Fig S4.** The contrast between different river typology trends in macroinvertebrate abundance change, for each trophic and taxonomic group, according to Tukey pairwise comparison tests. Darker colours show higher contrasts between river typologies, meaning there is a larger difference between their trends. Black dots show where the contrasts between trends are significant at the p≤0.05 level.


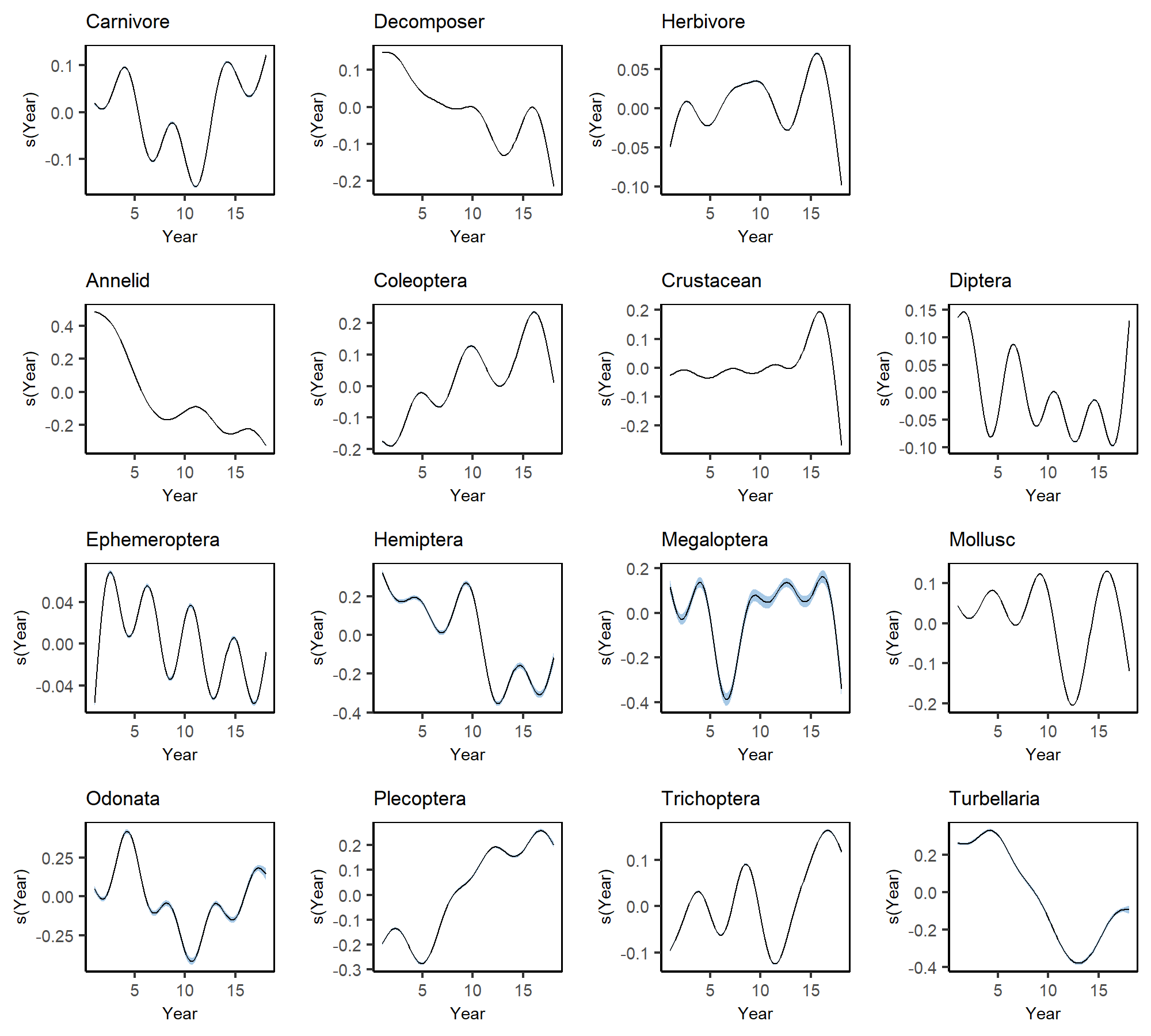


**Fig S5.** Plotted smooths of ‘Year’ fixed effects from GAMM trials, fitting site and observations as smoothed random effects in models. ‘Year’ is presented as integers 1-18 for the years 2002-2019. Approximate significance of smoothed terms can be found in Table S5.


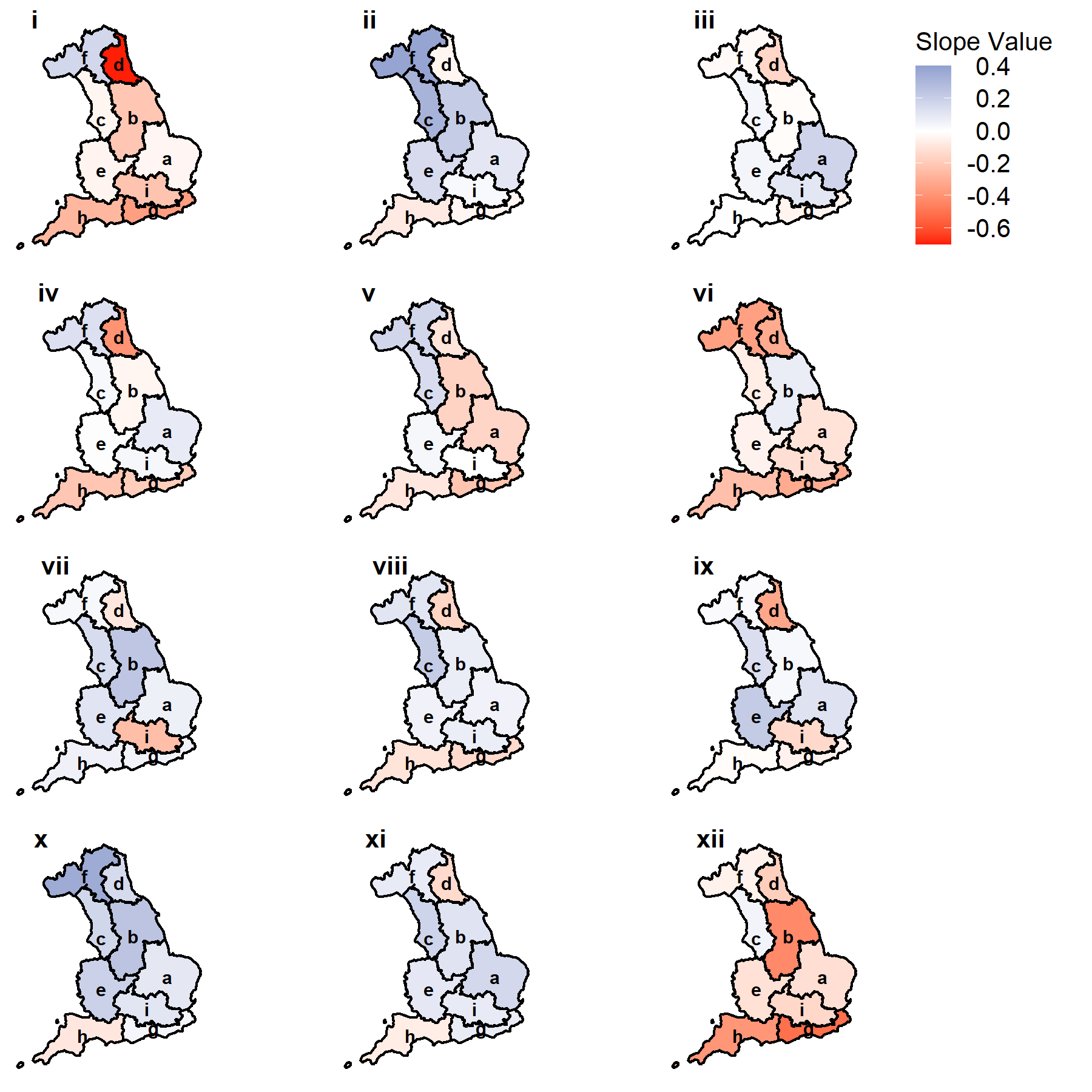


**Fig S6.** Map of English river basin district boundaries coloured by slope values extracted from models exploring spatial variation in trends, for i) annelids, ii) coleoptera, iii) crustaceans, iv) Diptera, v) Ephemeroptera, vi) Hemiptera, vii) Megaloptera, viii) molluscs, ix) Odonata, x) Plecoptera, xi) Trichoptera and xii) Turbellaria. Labels of river basins and the number of site:sample combinations are as follows; a) Anglian, n=12071; b) Humber; n=15396, c) North West, n=7519; d) Northumbria, n=3017; e) Severn, n=6727; f) Solway Tweed, 2101; g) South East, 4103; h) South West, n=7027, and i) Thames, n=8808. Map lines delineate study areas and do not necessarily depict accepted national boundaries.

**Supporting Tables**

**Table S1.** Anova outputs for model comparisons across each trophic and taxonomic group to test whether abundance trends varied significantly across river typologies. All model comparison outputs show that including river typology in the model improves the model significantly (p < 0.05). Model 1 and Model 2 include a ‘year’ effect only and a ‘year * typology interaction effect respectively.

| **Taxonomic**  **Group** | **AIC** | | **BIC** | | **Χ^2^** | **d.f.** | **P value** |
| --- | --- | --- | --- | --- | --- | --- | --- |
|  | **Model 1** | **Model 2** | **Model 1** | **Model 2** |  |  |  |
| Carnivore | 685760 | 685509 | 685824 | 685664 | 271 | 10 | ≤0.001 |
| Herbivore | 888601 | 888338 | 888665 | 888493 | 283 | 10 | ≤0.001 |
| Decomposer | 962839 | 962061 | 962903 | 962216 | 799 | 10 | ≤0.001 |
| Annelid | 703202 | 702302 | 703266 | 702457 | 921 | 10 | ≤0.001 |
| Coleoptera | 592003 | 591699 | 592067 | 591854 | 324 | 10 | ≤0.001 |
| Crustacean | 743626 | 741428 | 743690 | 741583 | 2218 | 10 | ≤0.001 |
| Diptera | 816119 | 815917 | 816183 | 816072 | 222 | 10 | ≤0.001 |
| Ephemeroptera | 743089 | 742327 | 743153 | 742482 | 782 | 10 | ≤0.001 |
| Hemiptera | 128902 | 128034 | 128965 | 128189 | 888 | 10 | ≤0.001 |
| Megaloptera | 92840 | 92370 | 92904 | 92525 | 490 | 10 | ≤0.001 |
| Mollusc | 715103 | 712865 | 715167 | 713021 | 2257 | 10 | ≤0.001 |
| Odonata | 140179 | 138864 | 140243 | 139019 | 1335 | 10 | ≤0.001 |
| Plecoptera | 285504 | 282614 | 285568 | 282769 | 2910 | 10 | ≤0.001 |
| Trichoptera | 683651 | 683325 | 683715 | 683480 | 346 | 10 | ≤0.001 |
| Turbellaria | 260237 | 260064 | 260301 | 260219 | 193 | 10 | ≤0.001 |

**Table S2.** Summary of trends across river typologies, for models including a year * typology effect across taxonomic and trophic. ‘AGR’ = Annual Growth Rate (%), and ‘% Change’ = Total percentage change over the 18 year time period.

| **Group** | **River Typology** | **Intercept** | | **Slope** | | | **AGR** | **% Change** |
| --- | --- | --- | --- | --- | --- | --- | --- | --- |
|  |  | **Estimate** | **S.E.** | **Estimate** | **S.E.** | **P value** |  |  |
| Annelid | Calcareous High | 3.429 | 0.069 | -0.041 | 0.006 | ≤0.001 | -2.78 | -50.0 |
|  | Calcareous Low | 4.101 | 0.045 | -0.026 | 0.004 | ≤0.001 | -1.99 | -35.9 |
|  | Organic High | 2.701 | 0.167 | -0.051 | 0.013 | ≤0.001 | -3.23 | -58.1 |
|  | Organic Low | 3.502 | 0.193 | -0.001 | 0.016 | 0.968 | -0.06 | -1.1 |
|  | Siliceous High | 3.462 | 0.069 | -0.057 | 0.006 | ≤0.001 | -3.45 | -62.0 |
|  | Siliceous Low | 4.39 | 0.068 | -0.073 | 0.006 | ≤0.001 | -3.96 | -71.3 |
| Coleoptera | Calcareous High | 2.532 | 0.106 | 0.059 | 0.007 | ≤0.001 | 9.62 | 173.1 |
|  | Calcareous Low | 2.257 | 0.062 | 0.021 | 0.005 | ≤0.001 | 2.40 | 43.3 |
|  | Organic High | 2.136 | 0.265 | 0.05 | 0.017 | ≤0.01 | 7.47 | 134.4 |
|  | Organic Low | 1.584 | 0.318 | 0.039 | 0.021 | 0.06 | 5.26 | 94.6 |
|  | Siliceous High | 2.824 | 0.106 | 0.029 | 0.007 | ≤0.001 | 3.56 | 64.1 |
|  | Siliceous Low | 3.549 | 0.103 | -0.012 | 0.007 | 0.1 | -1.03 | -18.5 |
| Crustacean | Calcareous High | 2.927 | 0.093 | -0.017 | 0.007 | <0.05 | -1.39 | -24.9 |
|  | Calcareous Low | 4.285 | 0.056 | 0.013 | 0.005 | ≤0.01 | 1.37 | 24.7 |
|  | Organic High | 0.597 | 0.244 | 0.004 | 0.017 | 0.808 | 0.40 | 7.3 |
|  | Organic Low | 4.128 | 0.269 | 0.034 | 0.019 | 0.075 | 4.32 | 77.7 |
|  | Siliceous High | 1.366 | 0.096 | -0.012 | 0.007 | 0.104 | -1.00 | -18.0 |
|  | Siliceous Low | 4.045 | 0.091 | -0.004 | 0.007 | 0.534 | -0.39 | -7.0 |
| Diptera | Calcareous High | 4.593 | 0.075 | -0.017 | 0.007 | ≤0.01 | -1.43 | -25.7 |
|  | Calcareous Low | 4.417 | 0.06 | 0.001 | 0.006 | 0.794 | 0.14 | 2.5 |
|  | Organic High | 4.007 | 0.148 | -0.016 | 0.012 | 0.17 | -1.34 | -24.1 |
|  | Organic Low | 3.348 | 0.172 | 0.032 | 0.015 | <0.05 | 4.05 | 72.9 |
|  | Siliceous High | 4.56 | 0.075 | -0.024 | 0.006 | ≤0.001 | -1.88 | -33.9 |
|  | Siliceous Low | 4.819 | 0.074 | -0.036 | 0.007 | ≤0.001 | -2.57 | -46.2 |
| Ephemeroptera | Calcareous High | 4.742 | 0.093 | 0.015 | 0.007 | <0.05 | 1.60 | 28.8 |
|  | Calcareous Low | 3.411 | 0.058 | -0.018 | 0.005 | ≤0.001 | -1.50 | -26.9 |
|  | Organic High | 4.117 | 0.228 | 0.015 | 0.015 | 0.308 | 1.61 | 29.0 |
|  | Organic Low | 2.799 | 0.274 | -0.038 | 0.02 | 0.052 | -2.65 | -47.7 |
|  | Siliceous High | 4.627 | 0.093 | -0.001 | 0.007 | 0.84 | -0.13 | -2.3 |
|  | Siliceous Low | 4.062 | 0.092 | -0.019 | 0.007 | ≤0.01 | -1.56 | -28.1 |
| Hemiptera | Calcareous High | -3.776 | 0.216 | -0.011 | 0.018 | 0.524 | -0.96 | -17.3 |
|  | Calcareous Low | -1.604 | 0.08 | -0.007 | 0.006 | 0.256 | -0.64 | -11.6 |
|  | Organic High | -3.883 | 0.675 | -0.11 | 0.066 | 0.095 | -4.70 | -84.6 |
|  | Organic Low | 0.63 | 0.454 | -0.02 | 0.029 | 0.495 | -1.59 | -28.5 |
|  | Siliceous High | -3.855 | 0.211 | 0.008 | 0.016 | 0.636 | 0.78 | 14.0 |
|  | Siliceous Low | -1.887 | 0.163 | -0.042 | 0.013 | ≤0.001 | -2.82 | -50.7 |
| Megaloptera | Calcareous High | -3.918 | 0.224 | 0.013 | 0.019 | 0.493 | 1.37 | 24.6 |
|  | Calcareous Low | -2.189 | 0.121 | 0 | 0.011 | 0.976 | 0.03 | 0.5 |
|  | Organic High | -3.304 | 0.496 | 0 | 0.039 | 0.992 | -0.04 | -0.7 |
|  | Organic Low | -1.566 | 0.477 | -0.089 | 0.04 | <0.05 | -4.33 | -78.0 |
|  | Siliceous High | -4.161 | 0.231 | 0.024 | 0.019 | 0.199 | 2.84 | 51.2 |
|  | Siliceous Low | -3.013 | 0.197 | 0.006 | 0.017 | 0.701 | 0.64 | 11.6 |
| Mollusc | Calcareous High | 2.155 | 0.101 | 0.012 | 0.008 | 0.142 | 1.28 | 23.0 |
|  | Calcareous Low | 4.052 | 0.074 | 0.012 | 0.007 | 0.074 | 1.23 | 22.1 |
|  | Organic High | 0.612 | 0.233 | 0.001 | 0.017 | 0.956 | 0.09 | 1.6 |
|  | Organic Low | 3.958 | 0.257 | 0.054 | 0.02 | ≤0.01 | 8.34 | 150.1 |
|  | Siliceous High | 1.702 | 0.102 | -0.019 | 0.008 | <0.05 | -1.52 | -27.4 |
|  | Siliceous Low | 3.868 | 0.099 | -0.007 | 0.008 | 0.37 | -0.66 | -11.8 |
| Odonata | Calcareous High | -4.978 | 0.295 | 0.047 | 0.024 | <0.05 | 6.81 | 122.6 |
|  | Calcareous Low | -1.578 | 0.182 | 0.014 | 0.016 | 0.379 | 1.53 | 27.6 |
|  | Organic High | -6.061 | 0.991 | 0.042 | 0.076 | 0.576 | 5.88 | 105.9 |
|  | Organic Low | 0.597 | 0.461 | 0.04 | 0.028 | 0.151 | 5.36 | 96.6 |
|  | Siliceous High | -3.987 | 0.265 | -0.018 | 0.022 | 0.418 | -1.44 | -25.9 |
|  | Siliceous Low | -1.098 | 0.219 | -0.012 | 0.018 | 0.505 | -1.02 | -18.3 |
| Plecoptera | Calcareous High | 1.63 | 0.169 | 0.056 | 0.009 | ≤0.001 | 8.83 | 159.0 |
|  | Calcareous Low | -2.697 | 0.106 | 0.062 | 0.008 | ≤0.001 | 10.44 | 187.9 |
|  | Organic High | 2.954 | 0.416 | 0.041 | 0.018 | <0.05 | 5.57 | 100.2 |
|  | Organic Low | -5.564 | 0.854 | 0.166 | 0.054 | ≤0.01 | 88.23 | 1588.2 |
|  | Siliceous High | 3.124 | 0.168 | 0.026 | 0.009 | ≤0.01 | 3.10 | 55.8 |
|  | Siliceous Low | 0.879 | 0.17 | 0.014 | 0.01 | 0.167 | 1.44 | 25.9 |
| Trichoptera | Calcareous High | 3.571 | 0.098 | 0.023 | 0.006 | ≤0.001 | 2.69 | 48.5 |
|  | Calcareous Low | 2.833 | 0.056 | 0.031 | 0.004 | ≤0.001 | 3.79 | 68.1 |
|  | Organic High | 3.308 | 0.249 | 0.012 | 0.014 | 0.387 | 1.29 | 23.2 |
|  | Organic Low | 1.421 | 0.302 | 0.073 | 0.018 | ≤0.001 | 13.63 | 245.4 |
|  | Siliceous High | 4.02 | 0.098 | -0.002 | 0.006 | 0.708 | -0.22 | -3.9 |
|  | Siliceous Low | 3.749 | 0.096 | 0.008 | 0.006 | 0.204 | 0.82 | 14.8 |
| Turbellaria | Calcareous High | -0.695 | 0.147 | -0.034 | 0.012 | ≤0.01 | -2.41 | -43.4 |
|  | Calcareous Low | 0 | 0.086 | -0.041 | 0.008 | ≤0.001 | -2.77 | -49.8 |
|  | Organic High | -1.532 | 0.396 | -0.016 | 0.031 | 0.604 | -1.31 | -23.6 |
|  | Organic Low | -0.631 | 0.415 | 0.11 | 0.032 | ≤0.001 | 30.53 | 549.5 |
|  | Siliceous High | 0.073 | 0.144 | -0.044 | 0.011 | ≤0.001 | -2.93 | -52.8 |
|  | Siliceous Low | 0.816 | 0.139 | -0.08 | 0.011 | ≤0.001 | -4.13 | -74.4 |
| Carnivore | Calcareous High | 3.504 | 0.076 | 0.021 | 0.006 | ≤0.001 | 2.44 | 44.0 |
|  | Calcareous Low | 3.38 | 0.058 | 0.012 | 0.005 | <0.05 | 1.27 | 22.9 |
|  | Organic High | 3.695 | 0.16 | 0.008 | 0.012 | 0.507 | 0.78 | 14.0 |
|  | Organic Low | 3.26 | 0.185 | 0.038 | 0.014 | ≤0.01 | 5.02 | 90.4 |
|  | Siliceous High | 3.999 | 0.076 | 0.003 | 0.006 | 0.668 | 0.26 | 4.7 |
|  | Siliceous Low | 3.87 | 0.075 | -0.009 | 0.006 | 0.15 | -0.80 | -14.4 |
| Herbivore | Calcareous High | 5.29 | 0.062 | 0.022 | 0.005 | ≤0.001 | 2.58 | 46.5 |
|  | Calcareous Low | 4.917 | 0.039 | 0.008 | 0.003 | <0.05 | 0.85 | 15.3 |
|  | Organic High | 5.054 | 0.151 | 0.005 | 0.011 | 0.627 | 0.50 | 9.1 |
|  | Organic Low | 4.558 | 0.178 | 0.035 | 0.013 | ≤0.01 | 4.60 | 82.8 |
|  | Siliceous High | 5.392 | 0.062 | 0.004 | 0.005 | 0.409 | 0.38 | 6.8 |
|  | Siliceous Low | 5.369 | 0.061 | -0.006 | 0.005 | 0.188 | -0.57 | -10.2 |
| Decomposer | Calcareous High | 5.66 | 0.052 | -0.021 | 0.004 | ≤0.001 | -1.64 | -29.6 |
|  | Calcareous Low | 5.97 | 0.035 | -0.005 | 0.003 | 0.092 | -0.47 | -8.5 |
|  | Organic High | 5.097 | 0.125 | -0.026 | 0.009 | ≤0.01 | -1.97 | -35.5 |
|  | Organic Low | 5.138 | 0.145 | 0.024 | 0.012 | <0.05 | 2.83 | 51.0 |
|  | Siliceous High | 5.644 | 0.052 | -0.039 | 0.004 | ≤0.001 | -2.68 | -48.2 |
|  | Siliceous Low | 6.298 | 0.052 | -0.039 | 0.004 | ≤0.001 | -2.68 | -48.2 |

**Table S3.** Summary of family-level trend slopes at the national level, basin effect across trophic groups. ‘AGR’ = Annual Growth Rate (%). We categorised trends as Strong Increases (where the annual growth rate ≥ 2.81%, leading to a doubling of abundance over 25 years); Moderate Increases (where the annual growth rate is between 1.16% and 2.81%); No Change (where trends were insignificant – all trends with growth rates between -1.14% and 1.16% were insignificant); Moderate Decreases (where the annual growth rate is between -2.73% and -1.14%); and Strong Decreases (where the annual growth rate ≤-2.73%, representing at least a halving of abundance over 25 years).

| **Group** | **Family** | **Slope** | **SE** | **P value** | **AGR (%)** | **Trend** |
| --- | --- | --- | --- | --- | --- | --- |
| Annelid | Erpobdellidae | -0.06 | 0.01 | ≤0.001 | -3.42 | Strong Decrease |
|  | Glossiphoniidae | 0.16 | 0.02 | ≤0.001 | 72.98 | Strong Increase |
|  | Piscicolidae | -0.07 | 0.01 | ≤0.001 | -3.80 | Strong Decrease |
|  | Lumbricidae | 0.06 | 0.02 | ≤0.01 | 10.00 | Strong Increase |
|  | Hirudinidae | -0.14 | 0.08 | 0.06 | -5.07 | No Change |
|  | Haemopidae | 0.02 | 0.05 | 0.70 | 2.32 | No Change |
|  | Lumbriculidae | -0.09 | 0.08 | 0.30 | -4.27 | No Change |
|  | Glossoscolecidae | -5.69 | 1.35 | ≤0.001 | -5.56 | Strong Decrease |
|  | Ampharetidae | 0.31 | 0.13 | <0.05 | 1080.08 | Strong Increase |
|  | Eunicidae | -0.95 | 1.39 | 0.49 | -5.56 | No Change |
|  | Trochochaetidae | -0.92 | 1.64 | 0.57 | -5.56 | No Change |
|  | Cirratulidae | -1.34 | 2.23 | 0.55 | -5.56 | No Change |
|  | Arenicolidae | -1.00 | 1.84 | 0.59 | -5.56 | No Change |
| Coleoptera | Dytiscidae | -0.02 | 0.01 | 0.06 | -1.66 | No Change |
|  | Elmidae | 0.05 | 0.01 | ≤0.001 | 7.01 | Strong Increase |
|  | Haliplidae | 0.15 | 0.02 | ≤0.001 | 61.60 | Strong Increase |
|  | Gyrinidae | 0.03 | 0.01 | <0.05 | 3.28 | Strong Increase |
|  | Scirtidae | 0.04 | 0.01 | ≤0.01 | 4.68 | Strong Increase |
|  | Hydrophilidae | -0.06 | 0.01 | ≤0.001 | -3.44 | Strong Decrease |
|  | Hygrobiidae | -0.08 | 0.05 | 0.11 | -4.22 | No Change |
|  | Hydraenidae | 0.02 | 0.01 | <0.05 | 2.92 | Strong Increase |
|  | Noteridae | -0.37 | 0.04 | ≤0.001 | -5.55 | Strong Decrease |
|  | Dryopidae | 0.07 | 0.02 | ≤0.001 | 13.41 | Strong Increase |
|  | Chrysomelidae | -0.02 | 0.02 | 0.36 | -1.65 | No Change |
|  | Curculionidae | -0.01 | 0.01 | 0.12 | -1.19 | No Change |
|  | Erirhinidae | -1.14 | 0.98 | 0.25 | -5.56 | No Change |
|  | Heteroceridae | -0.99 | 0.73 | 0.18 | -5.56 | No Change |
|  | Staphylinidae | 0.07 | 0.06 | 0.24 | 11.61 | No Change |
|  | Sphaeriusidae | -1.33 | 2.20 | 0.55 | -5.56 | No Change |
|  | Apionidae | -0.60 | 0.58 | 0.30 | -5.56 | No Change |
| Crustacean | Asellidae | -0.03 | 0.01 | <0.05 | -2.05 | Moderate Decrease |
|  | Crangonyctidae | 0.07 | 0.03 | ≤0.01 | 13.55 | Strong Increase |
|  | Astacidae | 0.13 | 0.02 | ≤0.001 | 41.40 | Strong Increase |
|  | Sphaeromatidae | -1.02 | 0.69 | 0.14 | -5.56 | No Change |
|  | Argulidae | -0.46 | 0.10 | ≤0.001 | -5.55 | Strong Decrease |
|  | Palaemonidae | -0.78 | 0.21 | ≤0.001 | -5.56 | Strong Decrease |
|  | Mysidae | -0.55 | 0.18 | ≤0.01 | -5.56 | Strong Decrease |
|  | Corophiidae | -0.61 | 0.08 | ≤0.001 | -5.56 | Strong Decrease |
|  | Niphargidae | 0.05 | 0.02 | <0.05 | 7.75 | Strong Increase |
|  | Daphniidae | -0.01 | 0.04 | 0.75 | -1.05 | No Change |
|  | Sididae | -0.66 | 0.55 | 0.23 | -5.56 | No Change |
|  | Notodelphyidae | -3.18 | 4.23 | 0.45 | -5.56 | No Change |
|  | Paguridae | -1.65 | 3.04 | 0.59 | -5.56 | No Change |
|  | Cambaridae | -2.11 | 3.48 | 0.54 | -5.56 | No Change |
|  | Chirocephalidae | -7.42 | 7.29 | 0.31 | -5.56 | No Change |
|  | Cercopagididae | -2.26 | 2.57 | 0.38 | -5.56 | No Change |
|  | Triopsidae | -1.17 | 1.87 | 0.53 | -5.56 | No Change |
|  | Janiridae | -15.68 | 38.82 | 0.69 | -5.56 | No Change |
|  | Bythocytheridae | -4.67 | 3.36 | 0.16 | -5.56 | No Change |
|  | Oplophoridae | -3.86 | 3.14 | 0.22 | -5.56 | No Change |
| Diptera | Chironomidae | -0.29 | 0.04 | ≤0.001 | -5.51 | Strong Decrease |
|  | Simuliidae | -0.02 | 0.01 | <0.05 | -1.92 | Moderate Decrease |
|  | Tipulidae | -0.13 | 0.02 | ≤0.001 | -4.93 | Strong Decrease |
|  | Ceratopogonidae | -0.02 | 0.01 | ≤0.01 | -1.91 | Moderate Decrease |
|  | Tabanidae | -0.02 | 0.01 | 0.11 | -1.58 | No Change |
|  | Pediciidae | 0.11 | 0.03 | ≤0.001 | 31.40 | Strong Increase |
|  | Limoniidae | 0.10 | 0.03 | ≤0.001 | 23.01 | Strong Increase |
|  | Psychodidae | 0.01 | 0.01 | 0.23 | 0.76 | No Change |
|  | Stratiomyidae | 0.01 | 0.01 | 0.59 | 0.53 | No Change |
|  | Empididae | 0.00 | 0.01 | 0.74 | -0.21 | No Change |
|  | Muscidae | 0.00 | 0.01 | 0.94 | 0.07 | No Change |
|  | Ptychopteridae | 0.08 | 0.02 | ≤0.001 | 15.51 | Strong Increase |
|  | Culicidae | -0.02 | 0.02 | 0.46 | -1.44 | No Change |
|  | Chaoboridae | -0.01 | 0.04 | 0.72 | -1.07 | No Change |
|  | Dixidae | 0.09 | 0.01 | ≤0.001 | 19.64 | Strong Increase |
|  | Ephydridae | 0.04 | 0.01 | ≤0.001 | 5.74 | Strong Increase |
|  | Syrphidae | 0.02 | 0.02 | 0.44 | 1.81 | No Change |
|  | Sciomyzidae | 0.03 | 0.02 | 0.20 | 3.68 | No Change |
|  | Dolichopodidae | 0.04 | 0.02 | <0.05 | 4.96 | Strong Increase |
|  | Rhagionidae | -0.03 | 0.02 | 0.10 | -2.16 | No Change |
|  | Thaumaleidae | -0.51 | 0.14 | ≤0.001 | -5.55 | Strong Decrease |
|  | Lonchopteridae | -0.58 | 0.17 | ≤0.001 | -5.56 | Strong Decrease |
|  | Bibionidae | 0.11 | 0.07 | 0.11 | 31.66 | No Change |
|  | Athericidae | 0.15 | 0.02 | ≤0.001 | 67.29 | Strong Increase |
|  | Scatopsidae | 0.13 | 0.07 | 0.07 | 42.54 | No Change |
| Ephemeroptera | Caenidae | -0.06 | 0.03 | <0.05 | -3.68 | Strong Decrease |
|  | Ephemeridae | 0.29 | 0.02 | ≤0.001 | 722.26 | Strong Increase |
|  | Baetidae | -0.03 | 0.02 | 0.06 | -2.24 | No Change |
|  | Ephemerellidae | 0.01 | 0.02 | 0.55 | 1.38 | No Change |
|  | Leptophlebiidae | 0.01 | 0.01 | 0.56 | 0.63 | No Change |
|  | Heptageniidae | 0.10 | 0.01 | ≤0.001 | 24.75 | Strong Increase |
|  | Plagiotropidaceae | 0.26 | 0.04 | ≤0.001 | 452.77 | Strong Increase |
|  | Siphlonuridae | -0.01 | 0.05 | 0.79 | -1.14 | No Change |
|  | Potamanthidae | -0.67 | 0.16 | ≤0.001 | -5.56 | Strong Decrease |
|  | Ameletidae | -0.40 | 0.09 | ≤0.001 | -5.55 | Strong Decrease |
| Hemiptera | Corixidae | -0.02 | 0.01 | <0.05 | -1.67 | Moderate Decrease |
|  | Notonectidae | -0.01 | 0.01 | 0.47 | -0.77 | No Change |
|  | Aphelocheiridae | -0.38 | 0.03 | ≤0.001 | -5.55 | Strong Decrease |
|  | Naucoridae | -0.33 | 0.04 | ≤0.001 | -5.53 | Strong Decrease |
|  | Veliidae | 0.01 | 0.01 | 0.62 | 0.50 | No Change |
|  | Gerridae | -0.02 | 0.01 | 0.13 | -1.42 | No Change |
|  | Pleidae | 0.05 | 0.03 | 0.12 | 6.53 | No Change |
|  | Nepidae | 0.00 | 0.01 | 0.83 | -0.30 | No Change |
|  | Hydrometridae | -0.10 | 0.02 | ≤0.001 | -4.55 | Strong Decrease |
|  | Mesoveliidae | 0.03 | 0.04 | 0.49 | 3.09 | No Change |
|  | Cicadellidae | -0.58 | 0.26 | <0.05 | -5.56 | Strong Decrease |
| Megaloptera | Sialidae | 0.01 | 0.01 | 0.51 | 0.79 | No Change |
| Mollusc | Lymnaeidae | -0.04 | 0.01 | ≤0.001 | -2.81 | Strong Decrease |
|  | Sphaeriidae | 0.01 | 0.01 | 0.34 | 0.88 | No Change |
|  | Bithyniidae | -0.05 | 0.02 | ≤0.01 | -3.37 | Strong Decrease |
|  | Valvatidae | -0.07 | 0.02 | ≤0.001 | -3.98 | Strong Decrease |
|  | Planorbidae | 0.05 | 0.01 | ≤0.001 | 7.81 | Strong Increase |
|  | Physidae | -0.01 | 0.01 | 0.41 | -0.73 | No Change |
|  | Hydrobiidae | -0.73 | 0.12 | ≤0.001 | -5.56 | Strong Decrease |
|  | Tateidae | 0.31 | 0.03 | ≤0.001 | 1006.03 | Strong Increase |
|  | Unionidae | -0.04 | 0.01 | ≤0.001 | -2.87 | Strong Decrease |
|  | Neritidae | -0.20 | 0.02 | ≤0.001 | -5.37 | Strong Decrease |
|  | Acroloxidae | 0.03 | 0.02 | 0.08 | 3.96 | No Change |
|  | Viviparidae | -0.37 | 0.03 | ≤0.001 | -5.54 | Strong Decrease |
|  | Dreissenidae | -0.47 | 0.05 | ≤0.001 | -5.55 | Strong Decrease |
|  | Succineidae | 0.06 | 0.01 | ≤0.001 | 10.42 | Strong Increase |
|  | Gastrodontidae | 0.05 | 0.03 | 0.08 | 8.50 | No Change |
|  | Cyrenidae | -0.57 | 0.61 | 0.34 | -5.56 | No Change |
|  | Donacidae | -0.69 | 1.36 | 0.61 | -5.56 | No Change |
|  | Carychiidae | -0.07 | 0.21 | 0.73 | -3.94 | No Change |
|  | Hygromiidae | -0.90 | 0.37 | <0.05 | -5.56 | Strong Decrease |
|  | Buccinidae | -1.84 | 2.70 | 0.50 | -5.56 | No Change |
|  | Columbellidae | -0.87 | 0.84 | 0.30 | -5.56 | No Change |
|  | Euconulidae | -2.44 | 1.33 | 0.07 | -5.56 | No Change |
|  | Caecidae | -3.98 | 2.67 | 0.14 | -5.56 | No Change |
|  | Lepidomeniidae | -2.39 | 2.68 | 0.37 | -5.56 | No Change |
|  | Tornidae | -0.07 | 0.21 | 0.73 | -3.94 | No Change |
|  | Tellinidae | -1.76 | 2.23 | 0.43 | -5.56 | No Change |
|  | Assimineidae | -0.83 | 3.87 | 0.83 | -5.56 | No Change |
|  | Cocculinidae | -4.01 | 3.34 | 0.23 | -5.56 | No Change |
|  | Ellobiidae | -1.50 | 3.43 | 0.66 | -5.56 | No Change |
|  | Pupillidae | -1.06 | 5.14 | 0.84 | -5.56 | No Change |
| Odonata | Calopterygidae | 0.03 | 0.02 | 0.06 | 3.80 | No Change |
|  | Platycnemididae | -0.09 | 0.02 | ≤0.001 | -4.34 | Strong Decrease |
|  | Coenagrionidae | -0.07 | 0.02 | ≤0.001 | -3.87 | Strong Decrease |
|  | Aeshnidae | -0.12 | 0.02 | ≤0.001 | -4.87 | Strong Decrease |
|  | Libellulidae | -0.01 | 0.03 | 0.67 | -1.20 | No Change |
|  | Gomphidae | -0.79 | 0.28 | ≤0.01 | -5.56 | Strong Decrease |
|  | Cordulegastridae | 0.16 | 0.01 | ≤0.001 | 76.35 | Strong Increase |
| Plecoptera | Nemouridae | 0.04 | 0.01 | ≤0.001 | 4.86 | Strong Increase |
|  | Perlodidae | 0.05 | 0.01 | ≤0.001 | 7.54 | Strong Increase |
|  | Leuctridae | 0.06 | 0.01 | ≤0.001 | 10.27 | Strong Increase |
|  | Taeniopterygidae | 0.02 | 0.02 | 0.25 | 2.46 | No Change |
|  | Chloroperlidae | 0.05 | 0.01 | ≤0.001 | 7.60 | Strong Increase |
|  | Perlidae | -0.25 | 0.02 | ≤0.001 | -5.48 | Strong Decrease |
|  | Capniidae | -0.01 | 0.05 | 0.77 | -1.17 | No Change |
| Trichoptera | Leptoceridae | 0.02 | 0.01 | <0.05 | 2.80 | Moderate Increase |
|  | Goeridae | 0.06 | 0.01 | ≤0.001 | 10.85 | Strong Increase |
|  | Limnephilidae | 0.05 | 0.01 | ≤0.001 | 6.50 | Strong Increase |
|  | Hydropsychidae | 0.01 | 0.01 | 0.34 | 0.91 | No Change |
|  | Sericostomatidae | 0.05 | 0.01 | ≤0.001 | 6.84 | Strong Increase |
|  | Molannidae | -0.06 | 0.01 | ≤0.001 | -3.39 | Strong Decrease |
|  | Polycentropodidae | 0.10 | 0.02 | ≤0.001 | 23.64 | Strong Increase |
|  | Glossosomatidae | 0.12 | 0.02 | ≤0.001 | 39.78 | Strong Increase |
|  | Psychomyiidae | 0.00 | 0.01 | 0.98 | -0.01 | No Change |
|  | Rhyacophilidae | 0.04 | 0.01 | ≤0.001 | 5.05 | Strong Increase |
|  | Lepidostomatidae | 0.06 | 0.01 | ≤0.001 | 11.20 | Strong Increase |
|  | Hydroptilidae | -0.01 | 0.01 | 0.45 | -0.83 | No Change |
|  | Phryganeidae | -0.07 | 0.01 | ≤0.001 | -3.79 | Strong Decrease |
|  | Beraeidae | 0.01 | 0.07 | 0.89 | 1.00 | No Change |
|  | Philopotamidae | 0.06 | 0.01 | ≤0.001 | 10.67 | Strong Increase |
|  | Apataniidae | -0.45 | 0.13 | ≤0.001 | -5.55 | Strong Decrease |
|  | Brachycentridae | 0.09 | 0.02 | ≤0.001 | 21.98 | Strong Increase |
|  | Ecnomidae | -0.61 | 0.18 | ≤0.001 | -5.56 | Strong Decrease |
|  | Odontoceridae | 0.04 | 0.01 | ≤0.001 | 5.97 | Strong Increase |
|  | Ichneumonidae | -1.08 | 1.14 | 0.34 | -5.56 | No Change |
| Turbellaria | Dugesiidae | 0.00 | 0.02 | 0.86 | -0.38 | No Change |
|  | Dendrocoelidae | -0.02 | 0.01 | 0.07 | -1.62 | No Change |
|  | Planariidae | -0.05 | 0.01 | ≤0.001 | -3.36 | Strong Decrease |
|  | Dalyelliidae | -8.77 | 20.97 | 0.68 | -5.56 | No Change |

**Table S4.** List of genera and families that we allocated to trophic groups according to data obtained on functional traits from Tachet et al. (2015).

| **Level of Identification** | **Group** | **Taxon** | **Dominant diet component** | **Trophic Group** |
| --- | --- | --- | --- | --- |
| Genus | Bivalvia | Corbicula | Living Microinvertebrates | Carnivore |
| Genus | Bivalvia | Congeria | Living Microinvertebrates | Carnivore |
| Genus | Bivalvia | Dreissena | Living Microinvertebrates | Carnivore |
| Genus | Bivalvia | Anodonta | Living Microinvertebrates | Carnivore |
| Genus | Bivalvia | Anodonta | Vertebrates | Carnivore |
| Genus | Bivalvia | Potomida (Psilunio) | Living Microinvertebrates | Carnivore |
| Genus | Bivalvia | Potomida (Psilunio) | Vertebrates | Carnivore |
| Genus | Bivalvia | Pseudanodonta | Living Microinvertebrates | Carnivore |
| Genus | Bivalvia | Pseudanodonta | Vertebrates | Carnivore |
| Genus | Bivalvia | Unio | Living Microinvertebrates | Carnivore |
| Genus | Bivalvia | Unio | Vertebrates | Carnivore |
| Genus | Coleoptera | Acilius | Living Macroinvertebrates | Carnivore |
| Genus | Coleoptera | Agabus | Living Macroinvertebrates | Carnivore |
| Genus | Coleoptera | Bidessus | Living Macroinvertebrates | Carnivore |
| Genus | Coleoptera | Colymbetes | Living Macroinvertebrates | Carnivore |
| Genus | Coleoptera | Copelatus | Living Macroinvertebrates | Carnivore |
| Genus | Coleoptera | Cybister | Living Macroinvertebrates | Carnivore |
| Genus | Coleoptera | Cybister | Vertebrates | Carnivore |
| Genus | Coleoptera | Deronectes | Living Macroinvertebrates | Carnivore |
| Genus | Coleoptera | Dytiscus | Living Macroinvertebrates | Carnivore |
| Genus | Coleoptera | Dytiscus | Vertebrates | Carnivore |
| Genus | Coleoptera | Eretes | Living Macroinvertebrates | Carnivore |
| Genus | Coleoptera | Graphoderus | Living Macroinvertebrates | Carnivore |
| Genus | Coleoptera | Graptodytes | Living Macroinvertebrates | Carnivore |
| Genus | Coleoptera | Hydaticus | Living Macroinvertebrates | Carnivore |
| Genus | Coleoptera | Hydroglyphus | Living Macroinvertebrates | Carnivore |
| Genus | Coleoptera | Hydroporus | Living Macroinvertebrates | Carnivore |
| Genus | Coleoptera | Hydrovatus | Living Macroinvertebrates | Carnivore |
| Genus | Coleoptera | Hygrotus | Living Macroinvertebrates | Carnivore |
| Genus | Coleoptera | Hyphydrus | Living Macroinvertebrates | Carnivore |
| Genus | Coleoptera | Ilybius | Living Macroinvertebrates | Carnivore |
| Genus | Coleoptera | Laccophilus | Living Macroinvertebrates | Carnivore |
| Genus | Coleoptera | Laccornis | Living Macroinvertebrates | Carnivore |
| Genus | Coleoptera | Meladema | Living Macroinvertebrates | Carnivore |
| Genus | Coleoptera | Metaporus | Living Macroinvertebrates | Carnivore |
| Genus | Coleoptera | Nebrioporus | Living Macroinvertebrates | Carnivore |
| Genus | Coleoptera | Oreodytes | Living Macroinvertebrates | Carnivore |
| Genus | Coleoptera | Platambus | Living Macroinvertebrates | Carnivore |
| Genus | Coleoptera | Porhydrus | Living Macroinvertebrates | Carnivore |
| Genus | Coleoptera | Rhantus | Living Macroinvertebrates | Carnivore |
| Genus | Coleoptera | Scarodytes | Living Macroinvertebrates | Carnivore |
| Genus | Coleoptera | Siettitia | Living Macroinvertebrates | Carnivore |
| Genus | Coleoptera | Stictonectes | Living Macroinvertebrates | Carnivore |
| Genus | Coleoptera | Stictotarsus | Living Macroinvertebrates | Carnivore |
| Genus | Coleoptera | Yola | Living Macroinvertebrates | Carnivore |
| Genus | Coleoptera | Aulonogyrus | Living Macroinvertebrates | Carnivore |
| Genus | Coleoptera | Gyrinus | Living Macroinvertebrates | Carnivore |
| Genus | Coleoptera | Orectochilus | Living Macroinvertebrates | Carnivore |
| Genus | Coleoptera | Anacaena | Living Microinvertebrates | Carnivore |
| Genus | Coleoptera | Berosus | Living Microinvertebrates | Carnivore |
| Genus | Coleoptera | Chaetarthria | Living Microinvertebrates | Carnivore |
| Genus | Coleoptera | Crenitis | Living Microinvertebrates | Carnivore |
| Genus | Coleoptera | Cymbiodyta | Living Microinvertebrates | Carnivore |
| Genus | Coleoptera | Enochrus | Living Microinvertebrates | Carnivore |
| Genus | Coleoptera | Helochares | Living Microinvertebrates | Carnivore |
| Genus | Coleoptera | Hemisphaera | Living Microinvertebrates | Carnivore |
| Genus | Coleoptera | Hydrobius | Living Microinvertebrates | Carnivore |
| Genus | Coleoptera | Hydrochara | Living Macroinvertebrates | Carnivore |
| Genus | Coleoptera | Hydrophilus | Living Macroinvertebrates | Carnivore |
| Genus | Coleoptera | Laccobius | Living Microinvertebrates | Carnivore |
| Genus | Coleoptera | Limnoxenus | Living Microinvertebrates | Carnivore |
| Genus | Coleoptera | Paracymus | Living Microinvertebrates | Carnivore |
| Genus | Coleoptera | Hygrobia | Living Macroinvertebrates | Carnivore |
| Genus | Coleoptera | Hygrobia | Living Microinvertebrates | Carnivore |
| Genus | Coleoptera | Noterus | Living Macroinvertebrates | Carnivore |
| Genus | Crustacea | Argulus | Vertebrates | Carnivore |
| Genus | Crustacea | Astacus | Living Macroinvertebrates | Carnivore |
| Genus | Crustacea | Astacus | Vertebrates | Carnivore |
| Genus | Crustacea | Orconectes | Living Macroinvertebrates | Carnivore |
| Genus | Crustacea | Dikerogammarus | Living Macroinvertebrates | Carnivore |
| Genus | Crustacea | Hemimysis | Living Microinvertebrates | Carnivore |
| Genus | Diptera | Atherix | Living Macroinvertebrates | Carnivore |
| Genus | Diptera | Atrichops | Living Macroinvertebrates | Carnivore |
| Genus | Diptera | Ceratopogoninae/Palpomyiinae | Living Microinvertebrates | Carnivore |
| Genus | Diptera | Chaoborus | Living Microinvertebrates | Carnivore |
| Genus | Diptera | Mochlonyx | Living Microinvertebrates | Carnivore |
| Genus | Diptera | Podonominae | Living Microinvertebrates | Carnivore |
| Genus | Diptera | Tanypodinae | Living Macroinvertebrates | Carnivore |
| Genus | Diptera | Anopheles | Living Microinvertebrates | Carnivore |
| Genus | Diptera | Culicinae | Living Microinvertebrates | Carnivore |
| Genus | Diptera | Dixella | Living Microinvertebrates | Carnivore |
| Genus | Diptera | Dolichopodidae | Living Macroinvertebrates | Carnivore |
| Genus | Diptera | Clinocerinae | Living Macroinvertebrates | Carnivore |
| Genus | Diptera | Hemerodromiinae | Living Macroinvertebrates | Carnivore |
| Genus | Diptera | Hexatomini | Living Macroinvertebrates | Carnivore |
| Genus | Diptera | Muscidae | Living Macroinvertebrates | Carnivore |
| Genus | Diptera | Pediciini | Living Macroinvertebrates | Carnivore |
| Genus | Diptera | Chrysopilus | Living Macroinvertebrates | Carnivore |
| Genus | Diptera | Sciomyzidae | Living Macroinvertebrates | Carnivore |
| Genus | Diptera | Syrphidae | Dead Animal | Carnivore |
| Genus | Diptera | Tabanidae | Living Macroinvertebrates | Carnivore |
| Genus | Ephemeroptera | Raptobaetopus | Living Macroinvertebrates | Carnivore |
| Genus | Gastropoda | Lymnaea | Dead Animal | Carnivore |
| Genus | Gastropoda | Ferrissia | Dead Animal | Carnivore |
| Genus | Heteroptera | Aphelocheirus | Living Macroinvertebrates | Carnivore |
| Genus | Heteroptera | Arctocorisa | Living Microinvertebrates | Carnivore |
| Genus | Heteroptera | Callicorixa | Living Microinvertebrates | Carnivore |
| Genus | Heteroptera | Corixa | Living Microinvertebrates | Carnivore |
| Genus | Heteroptera | Cymatia | Living Microinvertebrates | Carnivore |
| Genus | Heteroptera | Glaenocorisa | Living Microinvertebrates | Carnivore |
| Genus | Heteroptera | Micronecta | Living Microinvertebrates | Carnivore |
| Genus | Heteroptera | Paracorixa | Living Microinvertebrates | Carnivore |
| Genus | Heteroptera | Parasigara | Living Microinvertebrates | Carnivore |
| Genus | Heteroptera | Gerris | Living Macroinvertebrates | Carnivore |
| Genus | Heteroptera | Hydrometra | Dead Animal | Carnivore |
| Genus | Heteroptera | Hydrometra | Living Microinvertebrates | Carnivore |
| Genus | Heteroptera | Mesovelia | Living Microinvertebrates | Carnivore |
| Genus | Heteroptera | Ilyocoris | Living Macroinvertebrates | Carnivore |
| Genus | Heteroptera | Ilyocoris | Living Microinvertebrates | Carnivore |
| Genus | Heteroptera | Naucoris | Living Macroinvertebrates | Carnivore |
| Genus | Heteroptera | Nepa | Living Macroinvertebrates | Carnivore |
| Genus | Heteroptera | Nepa | Vertebrates | Carnivore |
| Genus | Heteroptera | Ranatra | Living Macroinvertebrates | Carnivore |
| Genus | Heteroptera | Ranatra | Vertebrates | Carnivore |
| Genus | Heteroptera | Anisops | Living Macroinvertebrates | Carnivore |
| Genus | Heteroptera | Notonecta | Living Macroinvertebrates | Carnivore |
| Genus | Heteroptera | Notonecta | Living Microinvertebrates | Carnivore |
| Genus | Heteroptera | Nychia | Living Macroinvertebrates | Carnivore |
| Genus | Heteroptera | Plea | Living Microinvertebrates | Carnivore |
| Genus | Heteroptera | Microvelia | Living Microinvertebrates | Carnivore |
| Genus | Heteroptera | Velia | Living Macroinvertebrates | Carnivore |
| Genus | Hirudinea | Dina | Living Macroinvertebrates | Carnivore |
| Genus | Hirudinea | Erpobdella | Living Macroinvertebrates | Carnivore |
| Genus | Hirudinea | Trocheta | Living Macroinvertebrates | Carnivore |
| Genus | Hirudinea | Glossiphonia | Living Macroinvertebrates | Carnivore |
| Genus | Hirudinea | Helobdella | Living Macroinvertebrates | Carnivore |
| Genus | Hirudinea | Hemiclepsis | Vertebrates | Carnivore |
| Genus | Hirudinea | Placobdella | Vertebrates | Carnivore |
| Genus | Hirudinea | Theromyzon | Vertebrates | Carnivore |
| Genus | Hirudinea | Haemopis | Living Macroinvertebrates | Carnivore |
| Genus | Hirudinea | Hirudo | Vertebrates | Carnivore |
| Genus | Hirudinea | Piscicola | Vertebrates | Carnivore |
| Genus | Megaloptera | Sialis | Living Macroinvertebrates | Carnivore |
| Genus | Nematomorpha | Gordiidae | Living Macroinvertebrates | Carnivore |
| Genus | Nemertia | Prostoma | Living Macroinvertebrates | Carnivore |
| Genus | Odonata | Aeshna | Living Macroinvertebrates | Carnivore |
| Genus | Odonata | Anax | Living Macroinvertebrates | Carnivore |
| Genus | Odonata | Boyeria | Living Macroinvertebrates | Carnivore |
| Genus | Odonata | Brachytron | Living Macroinvertebrates | Carnivore |
| Genus | Odonata | Hemianax | Living Macroinvertebrates | Carnivore |
| Genus | Odonata | Calopteryx | Living Macroinvertebrates | Carnivore |
| Genus | Odonata | Ceriagrion | Living Macroinvertebrates | Carnivore |
| Genus | Odonata | Coenagrion | Living Macroinvertebrates | Carnivore |
| Genus | Odonata | Enallagma | Living Macroinvertebrates | Carnivore |
| Genus | Odonata | Erythromma | Living Macroinvertebrates | Carnivore |
| Genus | Odonata | Ischnura | Living Macroinvertebrates | Carnivore |
| Genus | Odonata | Nehalennia | Living Macroinvertebrates | Carnivore |
| Genus | Odonata | Pyrrhosoma | Living Macroinvertebrates | Carnivore |
| Genus | Odonata | Cordulegaster | Living Macroinvertebrates | Carnivore |
| Genus | Odonata | Cordulia | Living Macroinvertebrates | Carnivore |
| Genus | Odonata | Epitheca | Living Macroinvertebrates | Carnivore |
| Genus | Odonata | Marcromia | Living Macroinvertebrates | Carnivore |
| Genus | Odonata | Oxygastra | Living Macroinvertebrates | Carnivore |
| Genus | Odonata | Somatochlora | Living Macroinvertebrates | Carnivore |
| Genus | Odonata | Gomphus | Living Macroinvertebrates | Carnivore |
| Genus | Odonata | Onychogomphus | Living Macroinvertebrates | Carnivore |
| Genus | Odonata | Ophiogomphus | Living Macroinvertebrates | Carnivore |
| Genus | Odonata | Paragomphus | Living Macroinvertebrates | Carnivore |
| Genus | Odonata | Chalcolestes | Living Macroinvertebrates | Carnivore |
| Genus | Odonata | Lestes | Living Macroinvertebrates | Carnivore |
| Genus | Odonata | Sympecma | Living Macroinvertebrates | Carnivore |
| Genus | Odonata | Crocothemis | Living Macroinvertebrates | Carnivore |
| Genus | Odonata | Leucorrhinia | Living Macroinvertebrates | Carnivore |
| Genus | Odonata | Libellula | Living Macroinvertebrates | Carnivore |
| Genus | Odonata | Orthetrum | Living Macroinvertebrates | Carnivore |
| Genus | Odonata | Sympetrum | Living Macroinvertebrates | Carnivore |
| Genus | Odonata | Platycnemis | Living Macroinvertebrates | Carnivore |
| Genus | Odonata | Platycnemis | Living Microinvertebrates | Carnivore |
| Genus | Oligochaeta | Chaetogaster | Living Microinvertebrates | Carnivore |
| Genus | Planipennia | Osmylus | Living Macroinvertebrates | Carnivore |
| Genus | Planipennia | Sisyra | Living Macroinvertebrates | Carnivore |
| Genus | Plecoptera | Chloroperla | Living Macroinvertebrates | Carnivore |
| Genus | Plecoptera | Siphonoperla | Living Macroinvertebrates | Carnivore |
| Genus | Plecoptera | Xanthoperla | Living Macroinvertebrates | Carnivore |
| Genus | Plecoptera | Dinocras | Living Macroinvertebrates | Carnivore |
| Genus | Plecoptera | Eoperla | Living Macroinvertebrates | Carnivore |
| Genus | Plecoptera | Marthamea | Living Macroinvertebrates | Carnivore |
| Genus | Plecoptera | Perla | Living Macroinvertebrates | Carnivore |
| Genus | Plecoptera | Arcynopteryx | Living Macroinvertebrates | Carnivore |
| Genus | Plecoptera | Besdolus | Living Macroinvertebrates | Carnivore |
| Genus | Plecoptera | Dictyogenus | Living Macroinvertebrates | Carnivore |
| Genus | Plecoptera | Diura | Living Macroinvertebrates | Carnivore |
| Genus | Plecoptera | Isogenus | Living Macroinvertebrates | Carnivore |
| Genus | Plecoptera | Isoperla | Living Macroinvertebrates | Carnivore |
| Genus | Plecoptera | Perlodes | Living Macroinvertebrates | Carnivore |
| Genus | Trichoptera | Ecnomus | Living Microinvertebrates | Carnivore |
| Genus | Trichoptera | Cheumatopsyche | Living Microinvertebrates | Carnivore |
| Genus | Trichoptera | Hydropsyche | Living Microinvertebrates | Carnivore |
| Genus | Trichoptera | Molanna | Living Microinvertebrates | Carnivore |
| Genus | Trichoptera | Molannodes | Living Microinvertebrates | Carnivore |
| Genus | Trichoptera | Odontocerum | Living Microinvertebrates | Carnivore |
| Genus | Trichoptera | Agrypnia | Living Macroinvertebrates | Carnivore |
| Genus | Trichoptera | Oligotricha | Living Macroinvertebrates | Carnivore |
| Genus | Trichoptera | Phryganea | Living Microinvertebrates | Carnivore |
| Genus | Trichoptera | Trichostegia | Living Microinvertebrates | Carnivore |
| Genus | Trichoptera | Cyrnus | Living Microinvertebrates | Carnivore |
| Genus | Trichoptera | Holocentropus | Living Microinvertebrates | Carnivore |
| Genus | Trichoptera | Neureclipsis | Living Microinvertebrates | Carnivore |
| Genus | Trichoptera | Plectrocnemia | Living Macroinvertebrates | Carnivore |
| Genus | Trichoptera | Polycentropus | Living Macroinvertebrates | Carnivore |
| Genus | Trichoptera | Rhyacophila | Living Macroinvertebrates | Carnivore |
| Genus | Trichoptera | Rhyacophila (Hyperrhyacophila) | Living Macroinvertebrates | Carnivore |
| Genus | Trichoptera | Rhyacophila (Hyporhyacophila) | Living Macroinvertebrates | Carnivore |
| Genus | Trichoptera | Rhyacophila (Metarhyacophila) | Living Macroinvertebrates | Carnivore |
| Genus | Trichoptera | Rhyacophila (Pararhyacophila) | Living Macroinvertebrates | Carnivore |
| Genus | Turbellaria | Bdellocephala | Living Macroinvertebrates | Carnivore |
| Genus | Turbellaria | Dendrocoelum | Living Macroinvertebrates | Carnivore |
| Genus | Turbellaria | Dugesia | Living Macroinvertebrates | Carnivore |
| Genus | Turbellaria | Crenobia | Living Macroinvertebrates | Carnivore |
| Genus | Turbellaria | Phagocata | Living Macroinvertebrates | Carnivore |
| Genus | Turbellaria | Planaria | Living Macroinvertebrates | Carnivore |
| Genus | Turbellaria | Polycelis | Living Macroinvertebrates | Carnivore |
| Genus | Bivalvia | Corbicula | Detritus | Decomposer |
| Genus | Bivalvia | Congeria | Detritus | Decomposer |
| Genus | Bivalvia | Dreissena | Detritus | Decomposer |
| Genus | Bivalvia | Anodonta | Dead Plant | Decomposer |
| Genus | Bivalvia | Potomida (Psilunio) | Dead Plant | Decomposer |
| Genus | Bivalvia | Pseudanodonta | Dead Plant | Decomposer |
| Genus | Bivalvia | Unio | Dead Plant | Decomposer |
| Genus | Coleoptera | Dryops | Dead Plant | Decomposer |
| Genus | Coleoptera | Pomatinus | Dead Plant | Decomposer |
| Genus | Coleoptera | Potamophilus | Dead Plant | Decomposer |
| Genus | Crustacea | Asellus | Dead Plant | Decomposer |
| Genus | Crustacea | Proasellus | Dead Plant | Decomposer |
| Genus | Crustacea | Austropotamobius | Dead Plant | Decomposer |
| Genus | Crustacea | Orconectes | Dead Plant | Decomposer |
| Genus | Crustacea | Corophium | Dead Plant | Decomposer |
| Genus | Crustacea | Corophium | Detritus | Decomposer |
| Genus | Crustacea | Echinogammarus | Dead Plant | Decomposer |
| Genus | Crustacea | Gammarus | Dead Plant | Decomposer |
| Genus | Crustacea | Niphargus | Detritus | Decomposer |
| Genus | Crustacea | Jaera | Dead Plant | Decomposer |
| Genus | Crustacea | Jaera | Detritus | Decomposer |
| Genus | Crustacea | Orchestia | Dead Plant | Decomposer |
| Genus | Crustacea | Lepidurus | Dead Plant | Decomposer |
| Genus | Crustacea | Lepidurus | Detritus | Decomposer |
| Genus | Crustacea | Triops | Dead Plant | Decomposer |
| Genus | Crustacea | Triops | Detritus | Decomposer |
| Genus | Diptera | Chironomini | Detritus | Decomposer |
| Genus | Diptera | Tanytarsini | Detritus | Decomposer |
| Genus | Diptera | Anopheles | Detritus | Decomposer |
| Genus | Diptera | Culicinae | Detritus | Decomposer |
| Genus | Diptera | Eriopterini | Dead Plant | Decomposer |
| Genus | Diptera | Limoniini | Dead Plant | Decomposer |
| Genus | Diptera | Psychodidae | Dead Plant | Decomposer |
| Genus | Diptera | Ptychopteridae | Dead Plant | Decomposer |
| Genus | Diptera | Prosimuliini | Detritus | Decomposer |
| Genus | Diptera | Simuliini | Detritus | Decomposer |
| Genus | Diptera | Syrphidae | Dead Plant | Decomposer |
| Genus | Diptera | Tipulidae | Dead Plant | Decomposer |
| Genus | Ephemeroptera | Ameletus | Dead Plant | Decomposer |
| Genus | Ephemeroptera | Metreletus | Dead Plant | Decomposer |
| Genus | Ephemeroptera | Metreletus | Detritus | Decomposer |
| Genus | Ephemeroptera | Cloeon | Detritus | Decomposer |
| Genus | Ephemeroptera | Brachycercus | Detritus | Decomposer |
| Genus | Ephemeroptera | Caenis | Detritus | Decomposer |
| Genus | Ephemeroptera | Torleya | Detritus | Decomposer |
| Genus | Ephemeroptera | Ephemera | Detritus | Decomposer |
| Genus | Ephemeroptera | Ecdyonurus | Dead Plant | Decomposer |
| Genus | Ephemeroptera | Electrogena | Detritus | Decomposer |
| Genus | Ephemeroptera | Choroterpes | Detritus | Decomposer |
| Genus | Ephemeroptera | Habroleptoides | Detritus | Decomposer |
| Genus | Ephemeroptera | Habrophlebia | Dead Plant | Decomposer |
| Genus | Ephemeroptera | Leptophlebia | Detritus | Decomposer |
| Genus | Ephemeroptera | Paraleptophlebia | Detritus | Decomposer |
| Genus | Ephemeroptera | Thraulus | Detritus | Decomposer |
| Genus | Ephemeroptera | Potamanthus | Dead Plant | Decomposer |
| Genus | Ephemeroptera | Potamanthus | Detritus | Decomposer |
| Genus | Ephemeroptera | Siphlonurus | Detritus | Decomposer |
| Genus | Gastropoda | Belgrandia | Dead Plant | Decomposer |
| Genus | Gastropoda | Bythiospeum | Dead Plant | Decomposer |
| Genus | Gastropoda | Lithoglyphus | Dead Plant | Decomposer |
| Genus | Gastropoda | Potamopyrgus | Dead Plant | Decomposer |
| Genus | Gastropoda | Bathyomphalus | Detritus | Decomposer |
| Genus | Gastropoda | Ferrissia | Dead Plant | Decomposer |
| Genus | Gastropoda | Planorbarius | Dead Plant | Decomposer |
| Genus | Gastropoda | Planorbis | Dead Plant | Decomposer |
| Genus | Gastropoda | Viviparus | Detritus | Decomposer |
| Genus | Heteroptera | Callicorixa | Dead Plant | Decomposer |
| Genus | Heteroptera | Hesperocorixa | Dead Plant | Decomposer |
| Genus | Heteroptera | Sigara | Dead Plant | Decomposer |
| Genus | Oligochaeta | Haplotaxis | Detritus | Decomposer |
| Genus | Oligochaeta | Dendrobaena | Detritus | Decomposer |
| Genus | Oligochaeta | Eiseniella | Detritus | Decomposer |
| Genus | Oligochaeta | other Lumbriculidae | Detritus | Decomposer |
| Genus | Oligochaeta | Stylodrilus | Detritus | Decomposer |
| Genus | Oligochaeta | Dero / Aulophorus | Detritus | Decomposer |
| Genus | Oligochaeta | Nais | Detritus | Decomposer |
| Genus | Oligochaeta | Ophidonais | Detritus | Decomposer |
| Genus | Oligochaeta | Paranais | Detritus | Decomposer |
| Genus | Oligochaeta | Piguetiella | Detritus | Decomposer |
| Genus | Oligochaeta | Pristina/Pristinella | Detritus | Decomposer |
| Genus | Oligochaeta | Ripistes | Detritus | Decomposer |
| Genus | Oligochaeta | Specaria | Detritus | Decomposer |
| Genus | Oligochaeta | Uncinais | Detritus | Decomposer |
| Genus | Oligochaeta | Vejdovskiella | Detritus | Decomposer |
| Genus | Oligochaeta | Branchiura | Detritus | Decomposer |
| Genus | Oligochaeta | other Tubificidae (ASC = with capillary setae) | Detritus | Decomposer |
| Genus | Oligochaeta | other Tubificidae (SSC = without capillary setae) | Detritus | Decomposer |
| Genus | Oligochaeta | Potamothrix | Detritus | Decomposer |
| Genus | Plecoptera | Capnioneura | Dead Plant | Decomposer |
| Genus | Plecoptera | Capnopsis | Dead Plant | Decomposer |
| Genus | Plecoptera | Capnopsis | Detritus | Decomposer |
| Genus | Plecoptera | Leuctra | Dead Plant | Decomposer |
| Genus | Plecoptera | Pachyleuctra | Dead Plant | Decomposer |
| Genus | Plecoptera | Amphinemura | Dead Plant | Decomposer |
| Genus | Plecoptera | Nemoura | Dead Plant | Decomposer |
| Genus | Plecoptera | Nemurella | Dead Plant | Decomposer |
| Genus | Plecoptera | Protonemura | Dead Plant | Decomposer |
| Genus | Plecoptera | Taeniopteryx | Dead Plant | Decomposer |
| Genus | Plecoptera | Taeniopteryx | Detritus | Decomposer |
| Genus | Polychaeta | Hypania | Detritus | Decomposer |
| Genus | Trichoptera | Cheumatopsyche | Detritus | Decomposer |
| Genus | Trichoptera | Diplectrona | Detritus | Decomposer |
| Genus | Trichoptera | Allotrichia | Detritus | Decomposer |
| Genus | Trichoptera | Stactobiella | Dead Plant | Decomposer |
| Genus | Trichoptera | Crunoecia | Dead Plant | Decomposer |
| Genus | Trichoptera | Lasiocephala | Dead Plant | Decomposer |
| Genus | Trichoptera | Lepidostoma | Dead Plant | Decomposer |
| Genus | Trichoptera | Ceraclea | Detritus | Decomposer |
| Genus | Trichoptera | Erotesis | Detritus | Decomposer |
| Genus | Trichoptera | Triaenodes | Detritus | Decomposer |
| Genus | Trichoptera | Allogamus | Dead Plant | Decomposer |
| Genus | Trichoptera | Anabolia | Dead Plant | Decomposer |
| Genus | Trichoptera | Glyphotaelius | Dead Plant | Decomposer |
| Genus | Trichoptera | Halesus | Dead Plant | Decomposer |
| Genus | Trichoptera | Ironoquia | Dead Plant | Decomposer |
| Genus | Trichoptera | Melampophylax | Dead Plant | Decomposer |
| Genus | Trichoptera | Mesophylax | Dead Plant | Decomposer |
| Genus | Trichoptera | Nemotaulius | Dead Plant | Decomposer |
| Genus | Trichoptera | other Limnephilini | Dead Plant | Decomposer |
| Genus | Trichoptera | other Stenophylacini / Chaetopterygini | Dead Plant | Decomposer |
| Genus | Trichoptera | Parachiona | Dead Plant | Decomposer |
| Genus | Trichoptera | Phacopteryx | Dead Plant | Decomposer |
| Genus | Trichoptera | Chimarra | Detritus | Decomposer |
| Genus | Trichoptera | Wormaldia | Detritus | Decomposer |
| Genus | Trichoptera | Hagenella | Dead Plant | Decomposer |
| Genus | Trichoptera | Oligostomis | Dead Plant | Decomposer |
| Genus | Trichoptera | Lype | Dead Plant | Decomposer |
| Genus | Trichoptera | Paduniella | Detritus | Decomposer |
| Genus | Trichoptera | Notidobia | Dead Plant | Decomposer |
| Genus | Trichoptera | Oecismus | Dead Plant | Decomposer |
| Genus | Trichoptera | Schizopelex | Dead Plant | Decomposer |
| Genus | Trichoptera | Sericostoma | Dead Plant | Decomposer |
| Genus | Bivalvia | Corbicula | Living Microphytes | Herbivore |
| Genus | Bivalvia | Musculium | Living Microphytes | Herbivore |
| Genus | Bivalvia | Pisidium | Living Microphytes | Herbivore |
| Genus | Bivalvia | Sphaerium | Living Microphytes | Herbivore |
| Genus | Bivalvia | Anodonta | Living Microphytes | Herbivore |
| Genus | Bivalvia | Potomida (Psilunio) | Living Microphytes | Herbivore |
| Genus | Bivalvia | Pseudanodonta | Living Microphytes | Herbivore |
| Genus | Bivalvia | Unio | Living Microphytes | Herbivore |
| Genus | Coleoptera | Donacia | Living Macrophytes | Herbivore |
| Genus | Coleoptera | Macroplea | Living Macrophytes | Herbivore |
| Genus | Coleoptera | Macroplea | Living Microphytes | Herbivore |
| Genus | Coleoptera | Plateumaris | Living Macrophytes | Herbivore |
| Genus | Coleoptera | Dryops | Living Microphytes | Herbivore |
| Genus | Coleoptera | Pomatinus | Living Microphytes | Herbivore |
| Genus | Coleoptera | Dupophilus | Living Microphytes | Herbivore |
| Genus | Coleoptera | Elmis | Living Microphytes | Herbivore |
| Genus | Coleoptera | Esolus | Living Microphytes | Herbivore |
| Genus | Coleoptera | Limnius | Living Microphytes | Herbivore |
| Genus | Coleoptera | Macronychus | Living Microphytes | Herbivore |
| Genus | Coleoptera | Normandia | Living Microphytes | Herbivore |
| Genus | Coleoptera | Oulimnius | Living Microphytes | Herbivore |
| Genus | Coleoptera | Potamophilus | Living Microphytes | Herbivore |
| Genus | Coleoptera | Riolus | Living Microphytes | Herbivore |
| Genus | Coleoptera | Stenelmis | Living Microphytes | Herbivore |
| Genus | Coleoptera | Brychius | Living Macrophytes | Herbivore |
| Genus | Coleoptera | Brychius | Living Microphytes | Herbivore |
| Genus | Coleoptera | Haliplus | Living Macrophytes | Herbivore |
| Genus | Coleoptera | Haliplus | Living Microphytes | Herbivore |
| Genus | Coleoptera | Peltodytes | Living Macrophytes | Herbivore |
| Genus | Coleoptera | Peltodytes | Living Microphytes | Herbivore |
| Genus | Coleoptera | Helophorus | Living Microphytes | Herbivore |
| Genus | Coleoptera | Hydraena | Living Microphytes | Herbivore |
| Genus | Coleoptera | Limnebius | Living Microphytes | Herbivore |
| Genus | Coleoptera | Ochthebius | Living Microphytes | Herbivore |
| Genus | Coleoptera | Anacaena | Living Microphytes | Herbivore |
| Genus | Coleoptera | Berosus | Living Microphytes | Herbivore |
| Genus | Coleoptera | Chaetarthria | Living Microphytes | Herbivore |
| Genus | Coleoptera | Crenitis | Living Microphytes | Herbivore |
| Genus | Coleoptera | Cymbiodyta | Living Microphytes | Herbivore |
| Genus | Coleoptera | Enochrus | Living Microphytes | Herbivore |
| Genus | Coleoptera | Helochares | Living Microphytes | Herbivore |
| Genus | Coleoptera | Hemisphaera | Living Microphytes | Herbivore |
| Genus | Coleoptera | Hydrobius | Living Microphytes | Herbivore |
| Genus | Coleoptera | Laccobius | Living Microphytes | Herbivore |
| Genus | Coleoptera | Limnoxenus | Living Microphytes | Herbivore |
| Genus | Coleoptera | Paracymus | Living Microphytes | Herbivore |
| Genus | Coleoptera | Cyphon | Living Microphytes | Herbivore |
| Genus | Coleoptera | Elodes | Living Microphytes | Herbivore |
| Genus | Coleoptera | Hydrocyphon | Living Microphytes | Herbivore |
| Genus | Coleoptera | Microcara | Living Microphytes | Herbivore |
| Genus | Coleoptera | Scirtes | Living Microphytes | Herbivore |
| Genus | Crustacea | Pacifastacus | Living Macrophytes | Herbivore |
| Genus | Crustacea | Procambarus | Living Macrophytes | Herbivore |
| Genus | Crustacea | Jaera | Living Microphytes | Herbivore |
| Genus | Crustacea | Limnomysis | Living Microphytes | Herbivore |
| Genus | Crustacea | Orchestia | Living Macrophytes | Herbivore |
| Genus | Diptera | Dasyheleinae | Living Microphytes | Herbivore |
| Genus | Diptera | Forcipomyinae | Living Microphytes | Herbivore |
| Genus | Diptera | Leptoconopinae | Living Microphytes | Herbivore |
| Genus | Diptera | Orthocladiinae / Diamesinae/Prodiamesinae | Living Microphytes | Herbivore |
| Genus | Diptera | Anopheles | Living Microphytes | Herbivore |
| Genus | Diptera | Culicinae | Living Microphytes | Herbivore |
| Genus | Diptera | Phalacrocera | Living Macrophytes | Herbivore |
| Genus | Diptera | Triogma | Living Macrophytes | Herbivore |
| Genus | Diptera | Dixa | Living Microphytes | Herbivore |
| Genus | Diptera | Ephydridae | Living Microphytes | Herbivore |
| Genus | Diptera | Thaumaleidae | Living Microphytes | Herbivore |
| Genus | Ephemeroptera | Metreletus | Living Microphytes | Herbivore |
| Genus | Ephemeroptera | Acentrella | Living Microphytes | Herbivore |
| Genus | Ephemeroptera | Baetis | Living Microphytes | Herbivore |
| Genus | Ephemeroptera | Centroptilum | Living Microphytes | Herbivore |
| Genus | Ephemeroptera | Cloeon | Living Microphytes | Herbivore |
| Genus | Ephemeroptera | Procloeon | Living Microphytes | Herbivore |
| Genus | Ephemeroptera | Pseudocentroptilum | Living Microphytes | Herbivore |
| Genus | Ephemeroptera | Ephemerella / Serratella | Living Microphytes | Herbivore |
| Genus | Ephemeroptera | Electrogena | Living Microphytes | Herbivore |
| Genus | Ephemeroptera | Epeorus | Living Microphytes | Herbivore |
| Genus | Ephemeroptera | Heptagenia | Living Microphytes | Herbivore |
| Genus | Ephemeroptera | Rhithrogena | Living Microphytes | Herbivore |
| Genus | Ephemeroptera | Choroterpes | Living Microphytes | Herbivore |
| Genus | Ephemeroptera | Habroleptoides | Living Microphytes | Herbivore |
| Genus | Ephemeroptera | Habrophlebia | Living Microphytes | Herbivore |
| Genus | Ephemeroptera | Thraulus | Living Microphytes | Herbivore |
| Genus | Ephemeroptera | Siphlonurus | Living Macrophytes | Herbivore |
| Genus | Gastropoda | Acroloxus | Living Microphytes | Herbivore |
| Genus | Gastropoda | Bithynia | Living Microphytes | Herbivore |
| Genus | Gastropoda | Belgrandia | Living Macrophytes | Herbivore |
| Genus | Gastropoda | Bythinella | Living Macrophytes | Herbivore |
| Genus | Gastropoda | Bythiospeum | Living Macrophytes | Herbivore |
| Genus | Gastropoda | Lithoglyphus | Living Macrophytes | Herbivore |
| Genus | Gastropoda | Galba | Living Microphytes | Herbivore |
| Genus | Gastropoda | Lymnaea | Living Microphytes | Herbivore |
| Genus | Gastropoda | Myxas | Living Macrophytes | Herbivore |
| Genus | Gastropoda | Radix | Living Microphytes | Herbivore |
| Genus | Gastropoda | Stagnicola | Living Macrophytes | Herbivore |
| Genus | Gastropoda | Theodoxus | Living Microphytes | Herbivore |
| Genus | Gastropoda | Aplexa | Living Microphytes | Herbivore |
| Genus | Gastropoda | Physa | Living Microphytes | Herbivore |
| Genus | Gastropoda | Physella | Living Macrophytes | Herbivore |
| Genus | Gastropoda | Physella | Living Microphytes | Herbivore |
| Genus | Gastropoda | Ancylus | Living Microphytes | Herbivore |
| Genus | Gastropoda | Anisus | Living Microphytes | Herbivore |
| Genus | Gastropoda | Ferrissia | Living Microphytes | Herbivore |
| Genus | Gastropoda | Gyraulus | Living Microphytes | Herbivore |
| Genus | Gastropoda | Hippeutis | Living Macrophytes | Herbivore |
| Genus | Gastropoda | Menetus | Living Macrophytes | Herbivore |
| Genus | Gastropoda | Segmentina | Living Macrophytes | Herbivore |
| Genus | Gastropoda | Valvata | Living Microphytes | Herbivore |
| Genus | Heteroptera | Callicorixa | Living Microphytes | Herbivore |
| Genus | Heteroptera | Sigara | Living Microphytes | Herbivore |
| Genus | Heteroptera | Plea | Living Macrophytes | Herbivore |
| Genus | Oligochaeta | Amphichaeta | Living Microphytes | Herbivore |
| Genus | Oligochaeta | Dero / Aulophorus | Living Microphytes | Herbivore |
| Genus | Oligochaeta | Nais | Living Microphytes | Herbivore |
| Genus | Oligochaeta | Paranais | Living Microphytes | Herbivore |
| Genus | Oligochaeta | Pristina/Pristinella | Living Microphytes | Herbivore |
| Genus | Oligochaeta | Slavina | Living Microphytes | Herbivore |
| Genus | Oligochaeta | Specaria | Living Microphytes | Herbivore |
| Genus | Oligochaeta | Stylaria | Living Microphytes | Herbivore |
| Genus | Oligochaeta | Uncinais | Living Microphytes | Herbivore |
| Genus | Plecoptera | Capnia | Living Macrophytes | Herbivore |
| Genus | Plecoptera | Capnia | Living Microphytes | Herbivore |
| Genus | Plecoptera | Leuctra | Living Macrophytes | Herbivore |
| Genus | Plecoptera | Brachyptera | Living Microphytes | Herbivore |
| Genus | Plecoptera | Rhabdiopteryx | Living Macrophytes | Herbivore |
| Genus | Trichoptera | Apatania | Living Microphytes | Herbivore |
| Genus | Trichoptera | Beraea | Living Microphytes | Herbivore |
| Genus | Trichoptera | Beraeamyia | Living Microphytes | Herbivore |
| Genus | Trichoptera | Beraeodes | Living Microphytes | Herbivore |
| Genus | Trichoptera | Beraeodina | Living Microphytes | Herbivore |
| Genus | Trichoptera | Ernodes | Living Microphytes | Herbivore |
| Genus | Trichoptera | Brachycentrus | Living Macrophytes | Herbivore |
| Genus | Trichoptera | Micrasema | Living Macrophytes | Herbivore |
| Genus | Trichoptera | Agapetus | Living Microphytes | Herbivore |
| Genus | Trichoptera | Catagapetus | Living Microphytes | Herbivore |
| Genus | Trichoptera | Glossosoma | Living Microphytes | Herbivore |
| Genus | Trichoptera | Ptilocolepus | Living Macrophytes | Herbivore |
| Genus | Trichoptera | Synagapetus | Living Microphytes | Herbivore |
| Genus | Trichoptera | Goera | Living Microphytes | Herbivore |
| Genus | Trichoptera | Lithax | Living Microphytes | Herbivore |
| Genus | Trichoptera | Silo | Living Microphytes | Herbivore |
| Genus | Trichoptera | Silonella | Living Microphytes | Herbivore |
| Genus | Trichoptera | Cheumatopsyche | Living Microphytes | Herbivore |
| Genus | Trichoptera | Hydropsyche | Living Microphytes | Herbivore |
| Genus | Trichoptera | Agraylea | Living Macrophytes | Herbivore |
| Genus | Trichoptera | Hydroptila | Living Macrophytes | Herbivore |
| Genus | Trichoptera | Ithytrichia | Living Microphytes | Herbivore |
| Genus | Trichoptera | Orthotrichia | Living Macrophytes | Herbivore |
| Genus | Trichoptera | Oxyethira | Living Macrophytes | Herbivore |
| Genus | Trichoptera | Stactobia | Living Macrophytes | Herbivore |
| Genus | Trichoptera | Tricholeiochiton | Living Macrophytes | Herbivore |
| Genus | Trichoptera | Adicella | Living Macrophytes | Herbivore |
| Genus | Trichoptera | Athripsodes | Living Macrophytes | Herbivore |
| Genus | Trichoptera | Erotesis | Living Macrophytes | Herbivore |
| Genus | Trichoptera | Leptocerus | Living Macrophytes | Herbivore |
| Genus | Trichoptera | Mystacides | Living Macrophytes | Herbivore |
| Genus | Trichoptera | Oecetis | Living Macrophytes | Herbivore |
| Genus | Trichoptera | Setodes | Living Macrophytes | Herbivore |
| Genus | Trichoptera | Triaenodes | Living Macrophytes | Herbivore |
| Genus | Trichoptera | Ylodes | Living Macrophytes | Herbivore |
| Genus | Trichoptera | Anabolia | Living Macrophytes | Herbivore |
| Genus | Trichoptera | Anabolia | Living Microphytes | Herbivore |
| Genus | Trichoptera | Anomalopterygella | Living Microphytes | Herbivore |
| Genus | Trichoptera | Cryptothrix | Living Microphytes | Herbivore |
| Genus | Trichoptera | Drusus | Living Microphytes | Herbivore |
| Genus | Trichoptera | Ecclisopteryx | Living Microphytes | Herbivore |
| Genus | Trichoptera | Halesus | Living Macrophytes | Herbivore |
| Genus | Trichoptera | Hydatophylax | Living Macrophytes | Herbivore |
| Genus | Trichoptera | Melampophylax | Living Macrophytes | Herbivore |
| Genus | Trichoptera | Mesophylax | Living Macrophytes | Herbivore |
| Genus | Trichoptera | Metanoea | Living Microphytes | Herbivore |
| Genus | Trichoptera | other Stenophylacini / Chaetopterygini | Living Macrophytes | Herbivore |
| Genus | Trichoptera | Parachiona | Living Macrophytes | Herbivore |
| Genus | Trichoptera | Odontocerum | Living Macrophytes | Herbivore |
| Genus | Trichoptera | Chimarra | Living Microphytes | Herbivore |
| Genus | Trichoptera | Philopotamus | Living Microphytes | Herbivore |
| Genus | Trichoptera | Wormaldia | Living Microphytes | Herbivore |
| Genus | Trichoptera | Hagenella | Living Macrophytes | Herbivore |
| Genus | Trichoptera | Oligostomis | Living Macrophytes | Herbivore |
| Genus | Trichoptera | Phryganea | Living Microphytes | Herbivore |
| Genus | Trichoptera | Trichostegia | Living Microphytes | Herbivore |
| Genus | Trichoptera | Paduniella | Living Microphytes | Herbivore |
| Genus | Trichoptera | Psychomyia | Living Microphytes | Herbivore |
| Genus | Trichoptera | Tinodes | Living Microphytes | Herbivore |
| Genus | Trichoptera | Rhyacophila (Prosrhyacophila) | Living Microphytes | Herbivore |
| Genus | Trichoptera | Notidobia | Living Macrophytes | Herbivore |
| Genus | Trichoptera | Oecismus | Living Macrophytes | Herbivore |
| Genus | Trichoptera | Schizopelex | Living Macrophytes | Herbivore |
| Family | Bivalvia | Cyrenidae | Living Microinvertebrates | Carnivore |
| Family | Bivalvia | Dreissenidae | Living Microinvertebrates | Carnivore |
| Family | Bivalvia | Unionidae | Living Microinvertebrates | Carnivore |
| Family | Bivalvia | Unionidae | Vertebrates | Carnivore |
| Family | Coleoptera | Dytiscidae | Living Macroinvertebrates | Carnivore |
| Family | Coleoptera | Gyrinidae | Living Macroinvertebrates | Carnivore |
| Family | Coleoptera | Hydrophilidae | Living Microinvertebrates | Carnivore |
| Family | Coleoptera | Hygrobiidae | Living Macroinvertebrates | Carnivore |
| Family | Coleoptera | Hygrobiidae | Living Microinvertebrates | Carnivore |
| Family | Coleoptera | Noteridae | Living Macroinvertebrates | Carnivore |
| Family | Crustacea | Argulidae | Vertebrates | Carnivore |
| Family | Crustacea | Cambaridae | Living Macroinvertebrates | Carnivore |
| Family | Crustacea | Grapsidae | Living Macroinvertebrates | Carnivore |
| Family | Crustacea | Mysidae | Living Microinvertebrates | Carnivore |
| Family | Diptera | Athericidae | Living Macroinvertebrates | Carnivore |
| Family | Diptera | Chaoboridae | Living Microinvertebrates | Carnivore |
| Family | Diptera | Culicidae | Living Microinvertebrates | Carnivore |
| Family | Diptera | Rhagionidae | Living Macroinvertebrates | Carnivore |
| Family | Heteroptera | Aphelocheiridae | Living Macroinvertebrates | Carnivore |
| Family | Heteroptera | Corixidae | Living Microinvertebrates | Carnivore |
| Family | Heteroptera | Gerridae | Living Macroinvertebrates | Carnivore |
| Family | Heteroptera | Hydrometridae | Dead Animal | Carnivore |
| Family | Heteroptera | Hydrometridae | Living Microinvertebrates | Carnivore |
| Family | Heteroptera | Mesoveliidae | Living Microinvertebrates | Carnivore |
| Family | Heteroptera | Naucoridae | Living Macroinvertebrates | Carnivore |
| Family | Heteroptera | Naucoridae | Living Microinvertebrates | Carnivore |
| Family | Heteroptera | Nepidae | Living Macroinvertebrates | Carnivore |
| Family | Heteroptera | Nepidae | Vertebrates | Carnivore |
| Family | Heteroptera | Notonectidae | Living Macroinvertebrates | Carnivore |
| Family | Heteroptera | Notonectidae | Living Microinvertebrates | Carnivore |
| Family | Heteroptera | Pleidae | Living Microinvertebrates | Carnivore |
| Family | Heteroptera | Veliidae | Living Microinvertebrates | Carnivore |
| Family | Hirudinea | Erpobdellidae | Living Macroinvertebrates | Carnivore |
| Family | Hirudinea | Glossiphoniidae | Living Macroinvertebrates | Carnivore |
| Family | Hirudinea | Haemopidae | Living Macroinvertebrates | Carnivore |
| Family | Hirudinea | Hirudinidae | Vertebrates | Carnivore |
| Family | Hirudinea | Piscicolidae | Vertebrates | Carnivore |
| Family | Megaloptera | Sialidae | Living Macroinvertebrates | Carnivore |
| Family | Nemertia | Tetrastemmatidae | Living Macroinvertebrates | Carnivore |
| Family | Odonata | Aeshnidae | Living Macroinvertebrates | Carnivore |
| Family | Odonata | Calopterygidae | Living Macroinvertebrates | Carnivore |
| Family | Odonata | Coenagrionidae | Living Macroinvertebrates | Carnivore |
| Family | Odonata | Cordulegastridae | Living Macroinvertebrates | Carnivore |
| Family | Odonata | Corduliidae | Living Macroinvertebrates | Carnivore |
| Family | Odonata | Gomphidae | Living Macroinvertebrates | Carnivore |
| Family | Odonata | Lestidae | Living Macroinvertebrates | Carnivore |
| Family | Odonata | Libellulidae | Living Macroinvertebrates | Carnivore |
| Family | Odonata | Platycnemididae | Living Macroinvertebrates | Carnivore |
| Family | Odonata | Platycnemididae | Living Microinvertebrates | Carnivore |
| Family | Planipennia | Osmylidae | Living Macroinvertebrates | Carnivore |
| Family | Planipennia | Sisyridae | Living Macroinvertebrates | Carnivore |
| Family | Plecoptera | Chloroperlidae | Living Macroinvertebrates | Carnivore |
| Family | Plecoptera | Perlidae | Living Macroinvertebrates | Carnivore |
| Family | Plecoptera | Perlodidae | Living Macroinvertebrates | Carnivore |
| Family | Trichoptera | Ecnomidae | Living Microinvertebrates | Carnivore |
| Family | Trichoptera | Hydropsychidae | Living Microinvertebrates | Carnivore |
| Family | Trichoptera | Molannidae | Living Microinvertebrates | Carnivore |
| Family | Trichoptera | Odontoceridae | Living Microinvertebrates | Carnivore |
| Family | Trichoptera | Phryganeidae | Living Macroinvertebrates | Carnivore |
| Family | Trichoptera | Polycentropodidae | Living Macroinvertebrates | Carnivore |
| Family | Trichoptera | Rhyacophilidae | Living Macroinvertebrates | Carnivore |
| Family | Turbellaria | Dendrocoelidae | Living Macroinvertebrates | Carnivore |
| Family | Turbellaria | Dugesiidae | Living Macroinvertebrates | Carnivore |
| Family | Turbellaria | Planariidae | Living Macroinvertebrates | Carnivore |
| Family | Diptera | Dolichopodidae | Living Macroinvertebrates | Carnivore |
| Family | Diptera | Muscidae | Living Macroinvertebrates | Carnivore |
| Family | Diptera | Sciomyzidae | Living Macroinvertebrates | Carnivore |
| Family | Diptera | Syrphidae | Dead Animal | Carnivore |
| Family | Diptera | Tabanidae | Living Macroinvertebrates | Carnivore |
| Family | Bivalvia | Cyrenidae | Detritus | Decomposer |
| Family | Bivalvia | Dreissenidae | Detritus | Decomposer |
| Family | Bivalvia | Unionidae | Dead Plant | Decomposer |
| Family | Coleoptera | Dryopidae | Dead Plant | Decomposer |
| Family | Coleoptera | Spercheidae | Dead Plant | Decomposer |
| Family | Crustacea | Asellidae | Dead Plant | Decomposer |
| Family | Crustacea | Cambaridae | Dead Plant | Decomposer |
| Family | Crustacea | Corophiidae | Dead Plant | Decomposer |
| Family | Crustacea | Corophiidae | Detritus | Decomposer |
| Family | Crustacea | Crangonyctidae | Dead Plant | Decomposer |
| Family | Crustacea | Gammaridae | Detritus | Decomposer |
| Family | Crustacea | Janiridae | Dead Plant | Decomposer |
| Family | Crustacea | Janiridae | Detritus | Decomposer |
| Family | Crustacea | Talitridae | Dead Plant | Decomposer |
| Family | Diptera | Chironomidae | Detritus | Decomposer |
| Family | Diptera | Culicidae | Detritus | Decomposer |
| Family | Ephemeroptera | Ameletidae | Dead Plant | Decomposer |
| Family | Ephemeroptera | Caenidae | Detritus | Decomposer |
| Family | Ephemeroptera | Ephemeridae | Detritus | Decomposer |
| Family | Ephemeroptera | Leptophlebiidae | Detritus | Decomposer |
| Family | Ephemeroptera | Potamanthidae | Dead Plant | Decomposer |
| Family | Ephemeroptera | Potamanthidae | Detritus | Decomposer |
| Family | Ephemeroptera | Siphlonuridae | Detritus | Decomposer |
| Family | Gastropoda | Hydrobiidae | Dead Plant | Decomposer |
| Family | Gastropoda | Viviparidae | Detritus | Decomposer |
| Family | Oligochaeta | Haplotaxidae | Detritus | Decomposer |
| Family | Oligochaeta | Lumbricidae | Detritus | Decomposer |
| Family | Oligochaeta | Tubificidae | Detritus | Decomposer |
| Family | Plecoptera | Leuctridae | Dead Plant | Decomposer |
| Family | Plecoptera | Nemouridae | Dead Plant | Decomposer |
| Family | Plecoptera | Taeniopterygidae | Detritus | Decomposer |
| Family | Polychaeta | Ampharetidae | Detritus | Decomposer |
| Family | Trichoptera | Lepidostomatidae | Dead Plant | Decomposer |
| Family | Trichoptera | Limnephilidae | Dead Plant | Decomposer |
| Family | Trichoptera | Sericostomatidae | Dead Plant | Decomposer |
| Family | Diptera | Psychodidae | Dead Plant | Decomposer |
| Family | Diptera | Ptychopteridae | Dead Plant | Decomposer |
| Family | Diptera | Stratiomyidae | Dead Plant | Decomposer |
| Family | Diptera | Syrphidae | Dead Plant | Decomposer |
| Family | Diptera | Tipulidae | Dead Plant | Decomposer |
| Family | Oligochaeta | Enchytraeidae | Detritus | Decomposer |
| Family | Bivalvia | Cyrenidae | Living Microphytes | Herbivore |
| Family | Bivalvia | Sphaeriidae | Living Microphytes | Herbivore |
| Family | Bivalvia | Unionidae | Living Microphytes | Herbivore |
| Family | Coleoptera | Chrysomelidae | Living Macrophytes | Herbivore |
| Family | Coleoptera | Dryopidae | Living Microphytes | Herbivore |
| Family | Coleoptera | Elmidae | Living Microphytes | Herbivore |
| Family | Coleoptera | Haliplidae | Living Macrophytes | Herbivore |
| Family | Coleoptera | Haliplidae | Living Microphytes | Herbivore |
| Family | Coleoptera | Helophoridae | Living Microphytes | Herbivore |
| Family | Coleoptera | Hydraenidae | Living Microphytes | Herbivore |
| Family | Coleoptera | Hydrochidae | Living Microphytes | Herbivore |
| Family | Coleoptera | Scirtidae | Living Microphytes | Herbivore |
| Family | Coleoptera | Curculionidae | Living Macrophytes | Herbivore |
| Family | Crustacea | Astacidae | Living Macrophytes | Herbivore |
| Family | Crustacea | Grapsidae | Living Macrophytes | Herbivore |
| Family | Crustacea | Janiridae | Living Microphytes | Herbivore |
| Family | Crustacea | Talitridae | Living Macrophytes | Herbivore |
| Family | Diptera | Culicidae | Living Microphytes | Herbivore |
| Family | Diptera | Cylindrotomidae | Living Macrophytes | Herbivore |
| Family | Diptera | Dixidae | Living Microphytes | Herbivore |
| Family | Diptera | Ephydridae | Living Microphytes | Herbivore |
| Family | Diptera | Thaumaleidae | Living Microphytes | Herbivore |
| Family | Ephemeroptera | Baetidae | Living Microphytes | Herbivore |
| Family | Ephemeroptera | Ephemerellidae | Living Microphytes | Herbivore |
| Family | Ephemeroptera | Heptageniidae | Living Microphytes | Herbivore |
| Family | Ephemeroptera | Siphlonuridae | Living Macrophytes | Herbivore |
| Family | Gastropoda | Acroloxidae | Living Microphytes | Herbivore |
| Family | Gastropoda | Bithyniidae | Living Microphytes | Herbivore |
| Family | Gastropoda | Lymnaeidae | Living Microphytes | Herbivore |
| Family | Gastropoda | Neritidae | Living Microphytes | Herbivore |
| Family | Gastropoda | Physidae | Living Microphytes | Herbivore |
| Family | Gastropoda | Planorbidae | Living Microphytes | Herbivore |
| Family | Gastropoda | Valvatidae | Living Microphytes | Herbivore |
| Family | Heteroptera | Pleidae | Living Macrophytes | Herbivore |
| Family | Oligochaeta | Naididae | Living Microphytes | Herbivore |
| Family | Plecoptera | Capniidae | Living Macrophytes | Herbivore |
| Family | Plecoptera | Capniidae | Living Microphytes | Herbivore |
| Family | Plecoptera | Leuctridae | Living Macrophytes | Herbivore |
| Family | Trichoptera | Apataniidae | Living Microphytes | Herbivore |
| Family | Trichoptera | Beraeidae | Living Microphytes | Herbivore |
| Family | Trichoptera | Brachycentridae | Living Macrophytes | Herbivore |
| Family | Trichoptera | Glossosomatidae | Living Microphytes | Herbivore |
| Family | Trichoptera | Goeridae | Living Microphytes | Herbivore |
| Family | Trichoptera | Hydroptilidae | Living Macrophytes | Herbivore |
| Family | Trichoptera | Leptoceridae | Living Macrophytes | Herbivore |
| Family | Trichoptera | Odontoceridae | Living Macrophytes | Herbivore |
| Family | Trichoptera | Philopotamidae | Living Microphytes | Herbivore |
| Family | Trichoptera | Psychomyiidae | Living Microphytes | Herbivore |

**Table S5.** Summary of final model coefficients (approximate estimates for parametric and smoothed terms, standard error [SE] of parametric terms, approximate significance of smoothed terms, and Un-Biased Risk Estimator [UBRE]) for Generalised Additive Mixed effect Model (GAMM) trials for all taxa and trophic groups.

| **Group** | **Intercept**  **(Parametric term)** | **S.E.** | **S(Year) (Fixed effect)** | **P value** | **S(Site) (Random effect)** | **P value** | **S(OLRE)**  **(Random effect)** | **P value** | **UBRE** |
| --- | --- | --- | --- | --- | --- | --- | --- | --- | --- |
| **Carnivore** | 4.44 | 0.0007 | 8.89 | ≤0.001 | 0.99 | ≤0.001 | 0.99 | ≤0.001 | 107 |
| **Herbivore** | 5.94 | 0.0004 | 8.95 | ≤0.001 | 1.00 | ≤0.001 | 0.91 | ≤0.001 | 352 |
| **Decomposer** | 6.48 | 0.0003 | 8.87 | ≤0.001 | 0.99 | ≤0.001 | 0.99 | ≤0.001 | 749 |
| **Annelid** | 4.74 | 0.0007 | 8.75 | ≤0.001 | 0.99 | ≤0.001 | 1.00 | ≤0.001 | 211 |
| **Coleoptera** | 4.34 | 0.0008 | 8.84 | ≤0.001 | 1.00 | ≤0.001 | 1.00 | ≤0.001 | 122 |
| **Crustacean** | 5.36 | 0.0004 | 8.83 | ≤0.001 | 0.98 | ≤0.001 | 1.00 | ≤0.001 | 537 |
| **Diptera** | 5.53 | 0.0005 | 8.95 | ≤0.001 | 1.00 | ≤0.001 | 0.98 | ≤0.001 | 377 |
| **Ephemeroptera** | 5.22 | 0.0005 | 8.96 | ≤0.001 | 1.00 | ≤0.001 | 0.99 | ≤0.001 | 278 |
| **Hemiptera** | 1.22 | 0.0038 | 8.97 | ≤0.001 | 0.99 | ≤0.001 | 0.92 | ≤0.001 | 18 |
| **Megaloptera** | -0.31 | 0.0072 | 8.94 | ≤0.001 | 0.97 | ≤0.001 | 0.00 | ≤0.001 | 3 |
| **Mollusc** | 5.17 | 0.0005 | 8.79 | ≤0.001 | 1.00 | ≤0.001 | 0.98 | ≤0.001 | 419 |
| **Odonata** | 0.53 | 0.0050 | 8.93 | ≤0.001 | 0.98 | ≤0.001 | 0.99 | ≤0.001 | 7 |
| **Plecoptera** | 3.10 | 0.0014 | 8.95 | ≤0.001 | 0.97 | ≤0.001 | 0.95 | ≤0.001 | 74 |
| **Trichoptera** | 4.78 | 0.0006 | 8.98 | ≤0.001 | 0.99 | ≤0.001 | 0.71 | ≤0.001 | 179 |
| **Turbellaria** | 1.82 | 0.0026 | 8.36 | ≤0.001 | 0.97 | ≤0.001 | 1.00 | ≤0.001 | 31 |
